# Correction: Ahn, J.W., et al. CeRNA Network Analysis Representing Characteristics of Different Tumor Environments Based on 1p/19q Codeletion in Oligodendrogliomas. *Cancers* 2020, *12*, 2543

**DOI:** 10.3390/cancers13030478

**Published:** 2021-01-27

**Authors:** Ju Won Ahn, YoungJoon Park, Su Jung Kang, So Jung Hwang, Kyung Gi Cho, JaeJoon Lim, KyuBum Kwack

**Affiliations:** 1 Department of Biomedical Science, College of Life Science, CHA University, Seongnam 13488, Korea; eugene@chauniv.ac.kr (J.W.A.); yjparkep@chauniv.ac.kr (Y.P.); sujung_k@chauniv.ac.kr (S.J.K.); 2 Department of Neurosurgery, Bundang CHA Medical Center, CHA University School of Medicine, Seongnam 13496, Korea; sjhwang7@chamc.co.kr (S.J.H.); sandori50@chamc.co.kr (K.G.C.)

The authors wish to make the following corrections to this paper [1]:

In the original article, there were mistakes in Figure 3A, Figure 5B and Table 1 as published.

Firstly, the authors wrote incorrect units on the Y axis in Figure 3A. This was due to a mistake in the figure editing.

Thus, the original Figure 3A listed below:

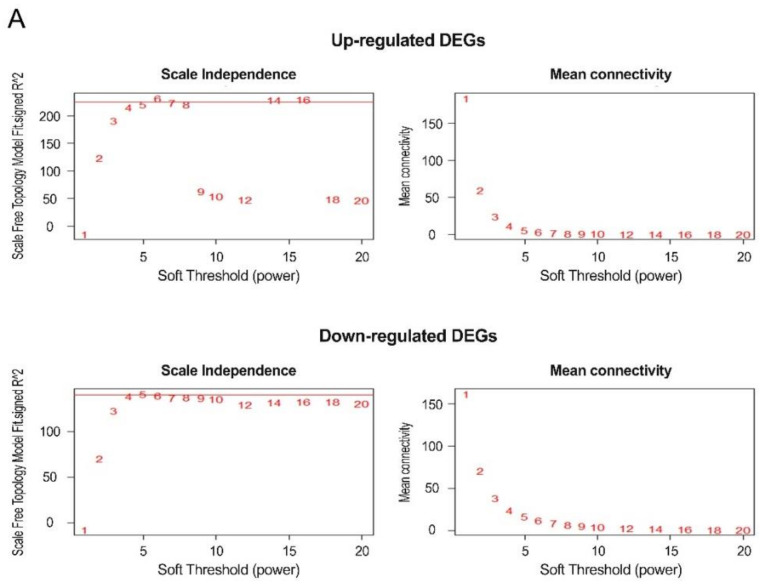



should be replaced with the following version:

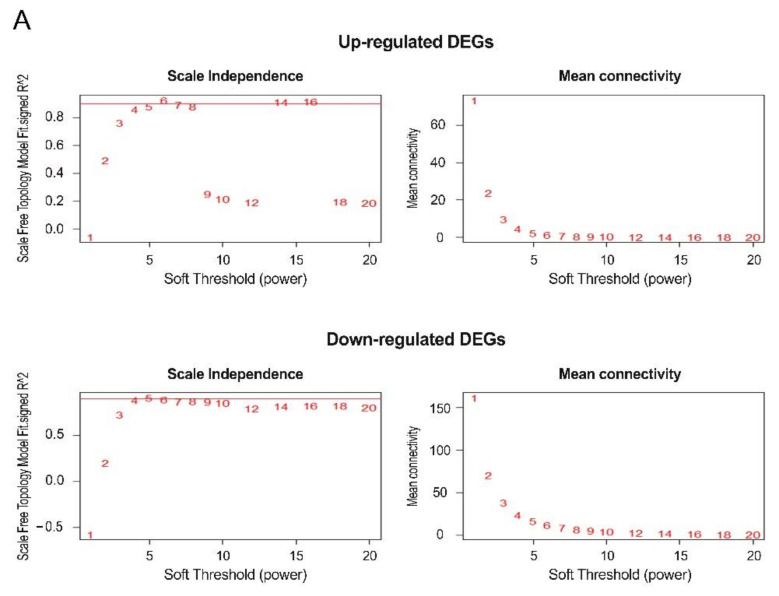



Secondly, the authors wrote p-value incorrectly in Figure 5B. This was also due to a mistake in the figure editing. Additionally, we would like to add the corresponding indication (“Table 2”) in result Section 3.4 and to replace the corresponding indication (“Figures 5B” to “Figure 5”) in discussion section to help our readers understand better.

The following sentence in result Section 3.4:

“In addition, in the data of patients extended to lower grade glioma, a higher number of non-coding RNAs in the ceRNA network showed a difference in OS rate according to the expression level.”

should be replaced with:

“In addition, in the data of patients extended to lower grade glioma, a higher number of non-coding RNAs in the ceRNA network showed a difference in OS rate according to the expression level (Table 2).”

The following sentence in the discussion section:

“Characteristically, the difference in survival rate among patients according to actual 1p/19q codeletion subtypes in the OD data of TCGA was not statistically significantly separated, but a difference of survival rate in the group as per the expression level of ceRNA was divided (Figure 5B).”

should be replaced with:

“Characteristically, the difference in survival rate among patients according to actual 1p/19q codeletion subtypes in the OD data of TCGA was not statistically significantly separated, but a difference of survival rate in the group as per the expression level of ceRNA was divided (Figure 5).”

Furthermore, the original Figure 5 listed below:

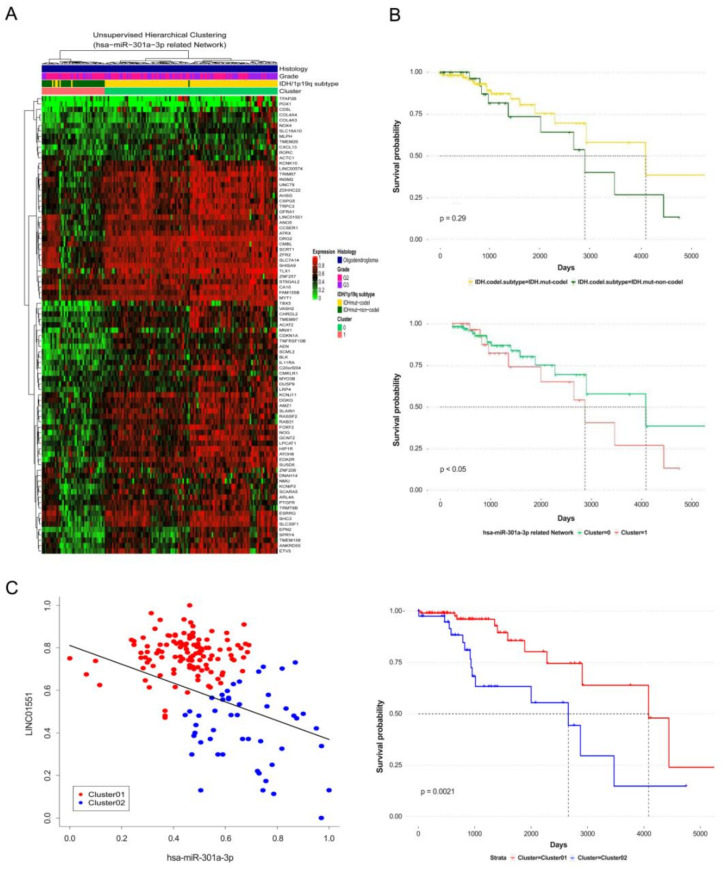



should be replaced with the following version:

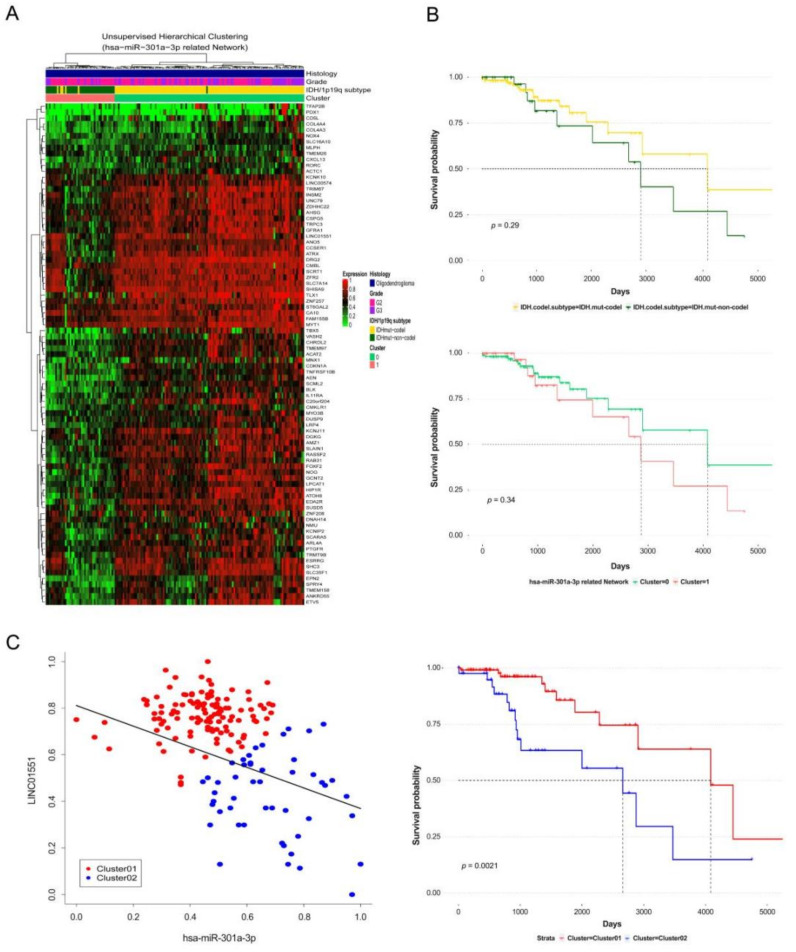



Finally, there was an alignment error in the results in Table 1, which was corrected. The values in Table 1 were misaligned; however, every network listed in Table 1 is the result of passing the threshold of the adjusted rand index (ARI), and since the correct information was already included in Table S1, the misaligned results do not affect our research results.

The original version of Table 1 is:

**Table 1:** Compare with Oligodenroglioma and Lower grade glioma using by adjusted rand index (ARI) evaluation of ceRNA network.

Network	Source miRNAs	No. Target Coding Gene	No. Target Non-Coding Gene	ARI	
				Oligodenroglioma (k* = 2)	Lower Grade Glioma (k* = 3)
Network01	hsa-miR-296-5p	78	6	0.872	0.724
Network02	hsa-miR-455-3p	68	1	0.692	0.671
Network03	hsa-miR-760	92	1	0.896	0.761
Network04	hsa-miR-1298-5p	43	4	0.871	0.747
Network05	hsa-miR-197-3p	78	5	0.872	0.770
Network06	hsa-miR-301a-5p	25	2	0.801	0.756
Network07	hsa-miR-1262	62	2	0.801	0.497
Network08	hsa-miR-186-5p	99	9	0.921	0.806
Network09	hsa-miR-301a-3p	83	2	0.847	0.834
Network10	hsa-miR-383-5p	97	1	0.872	0.728
Network11	hsa-miR-2114-3p	30	2	0.778	0.637
Network12	hsa-miR-204-5p	138	7	0.824	0.714
Network13	hsa-miR-7156-5p	31	6	0.896	0.888
Network14	hsa-miR-92b-3p	76	4	0.800	0.594
Network15	hsa-miR-3074-5p	31	9	0.896	0.789
Network16	hsa-miR-1298-3p	30	2	0.778	0.704

k*; Number of agglomerative cluster using for ARI.

should be replaced with the following Table 1:

The authors apologize for any inconvenience caused and state that the scientific conclusions are unaffected. The original article has been updated.

## Figures and Tables

**Table 1:** Comparison of oligodenroglioma and lower grade glioma using the adjusted rand index (ARI) evaluation of the ceRNA network.

Network	Source miRNAs	No. Target Coding Gene	No. Target Non-Coding Gene	ARI	
				Oligodenroglioma (k* = 2)	Lower Grade Glioma (k* = 3)
Network01	hsa-miR-296-5p	78	6	0.921	0.806
Network02	hsa-miR-455-3p	68	1	0.896	0.888
Network03	hsa-miR-760	92	1	0.896	0.789
Network04	hsa-miR-1298-5p	43	4	0.896	0.761
Network05	hsa-miR-197-3p	78	5	0.872	0.770
Network06	hsa-miR-301a-5p	25	2	0.872	0.728
Network07	hsa-miR-1262	62	2	0.872	0.724
Network08	hsa-miR-186-5p	99	9	0.871	0.747
Network09	hsa-miR-301a-3p	83	2	0.847	0.834
Network10	hsa-miR-383-5p	97	1	0.824	0.714
Network11	hsa-miR-2114-3p	30	2	0.801	0.497
Network12	hsa-miR-204-5p	138	7	0.801	0.756
Network13	hsa-miR-7156-5p	31	6	0.800	0.594
Network14	hsa-miR-92b-3p	76	4	0.778	0.704
Network15	hsa-miR-3074-5p	31	9	0.778	0.637
Network16	hsa-miR-1298-3p	30	2	0.692	0.671

k*; The number of agglomerative clusters using the ARI.
