# Supplementary material for: CeRNA Network Analysis Representing Characteristics of Different Tumor Environments Based on 1p/19q Codeletion in Oligodendrogliomas
Source: Cancers (Basel). 2020 Sep 7;12(9):2543. doi: 10.3390/cancers12092543 (PMC7564449; doi:10.3390/cancers12092543)
Supplement: Supplementary file 1 [file cancers-12-02543-s001.pdf]

Supplementary Materials: CeRNA Network Analysis Representing Characteristics of Different Tumor Environments Based on 1p/19q Codeletion in Oligodendrogliomas

Ju Won Ahn, YoungJoon Park, Su Jung Kang, So Jung Hwang, Kyung Gi Cho, JaeJoon Lim and KyuBum Kwack

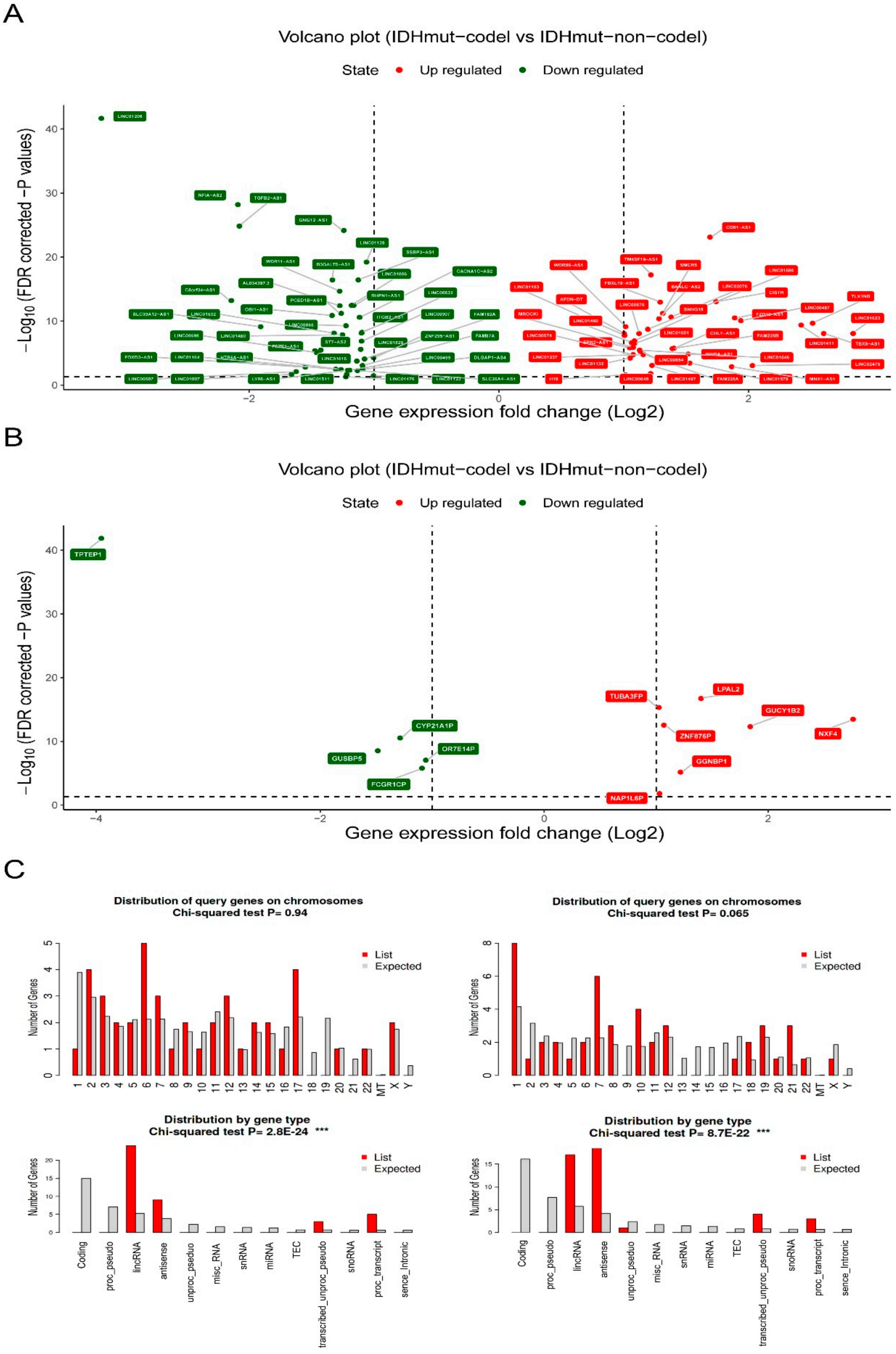

Figure S1. Volcano plot and distribution of gene on chromosomes for DEncRNAs between OD 1p/19q codeletion group and non-codeletion group. The volcano plot of DElncRNAs (**A**) and DEpseudogenes (**B**). The red dots represent significantly upregulated genes, the green dots represent significantly

downregulated genes (|log2 FC| ≥ 1 and FDR < 0.05). Histogram of input genetype and distribution on chromosomes for upregulated DEncRNAs (**left**) and downregulated DEncRNAs (**right**) (**C**).

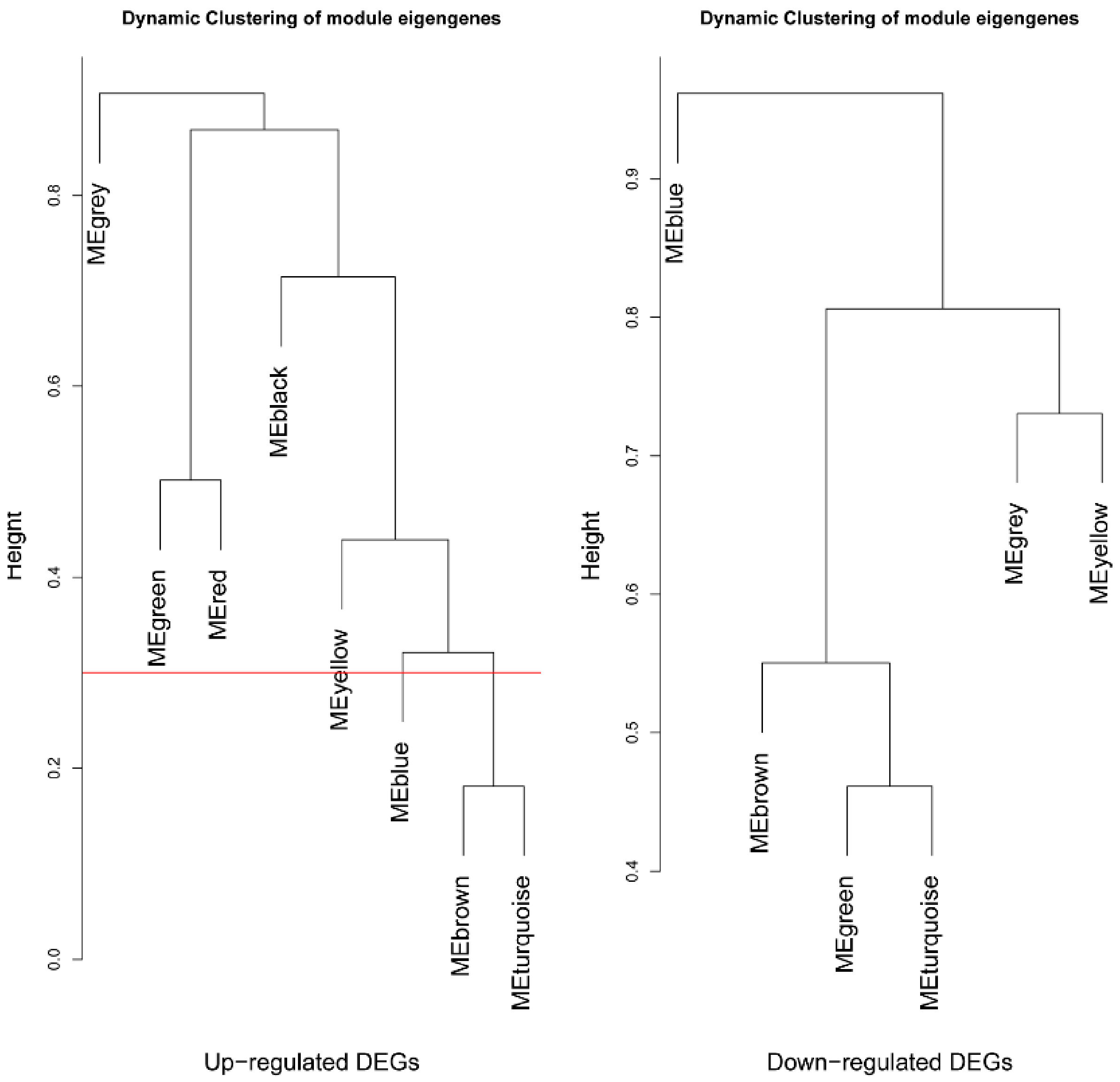

Figure S2. Dendrogram for merging modules by dynamic tree cut algorithm. The calculation of threshold for merging were based on correlation interconnectivity among modules.

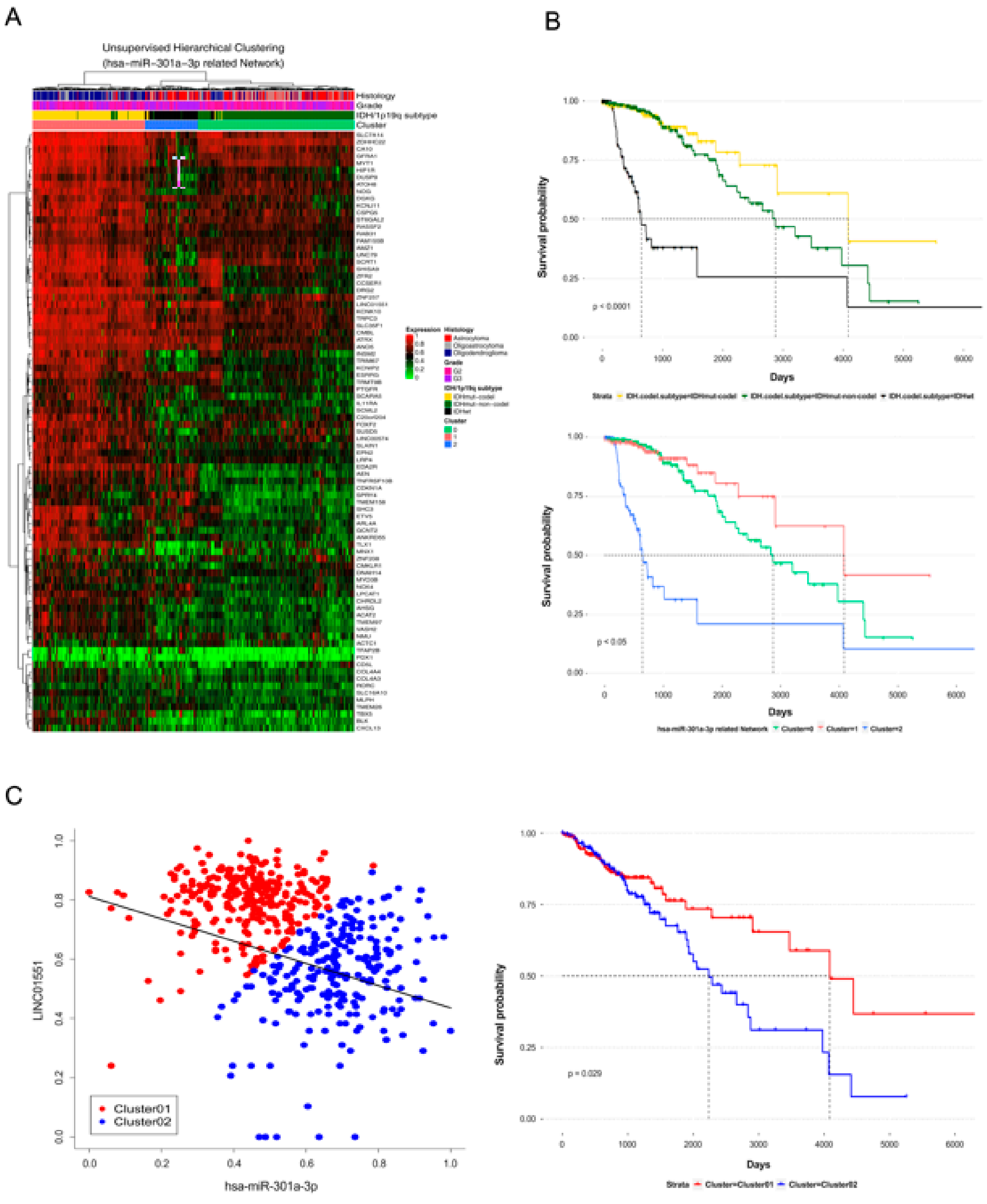

Figure S3. Expanded evaluation of ceRNA network through by unsupervised clustering and comparing survival to actual LGG patients from TCGA database. Heatmap for unsupervised analysis of DEcodingRNAs, DEncRNAs related to hsa-miR-301a-3p in ceRNA network (**A**). Kaplan Meier plot for comparing survival to between actual LGG patients including *IDH* wildtype from TCGA database and unsupervised group (**B**). Yellow line indicates survival data for LGG patients with 1p/19q codeletion, deep green line for LGG patients with 1p/19q non-codeletion and black line indicate for LGG patient with *IDH* wildtype (**Top**). Olive green, coral pink and skyblue line indicate groups from unsupervised clustering analysis with miR-301a-3p related ceRNA network. Scatter plot for clustering group by K-means in overall LGG patients (**Bottom**) (**C**). Scatter plot resenting negative correlation between

LINC01551 expected with ceRNA and related to miRNA in ceRNA network (**left**). Kaplan Meier survival plot according to the expression levels of ceRNA and miRNA (**right**).

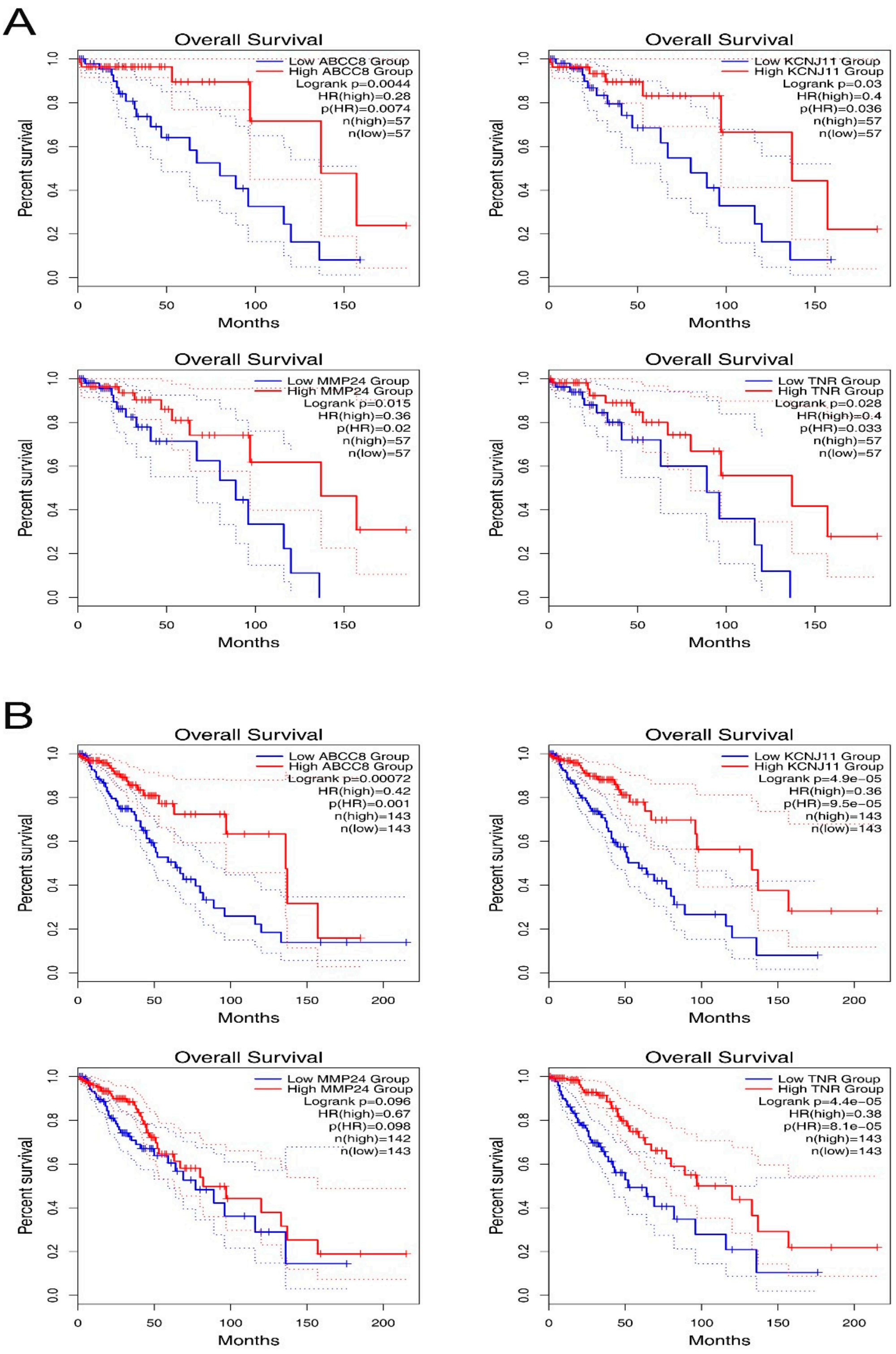

Figure S4. Kaplan Meier survival plot on *ABCC8, KCNJ11, MMP24, TNR* in OD patients (**A**). Kaplan Meier survival plot on *ABCC8, KCNJ11, MMP24, TNR* in LGG patients (**B**).

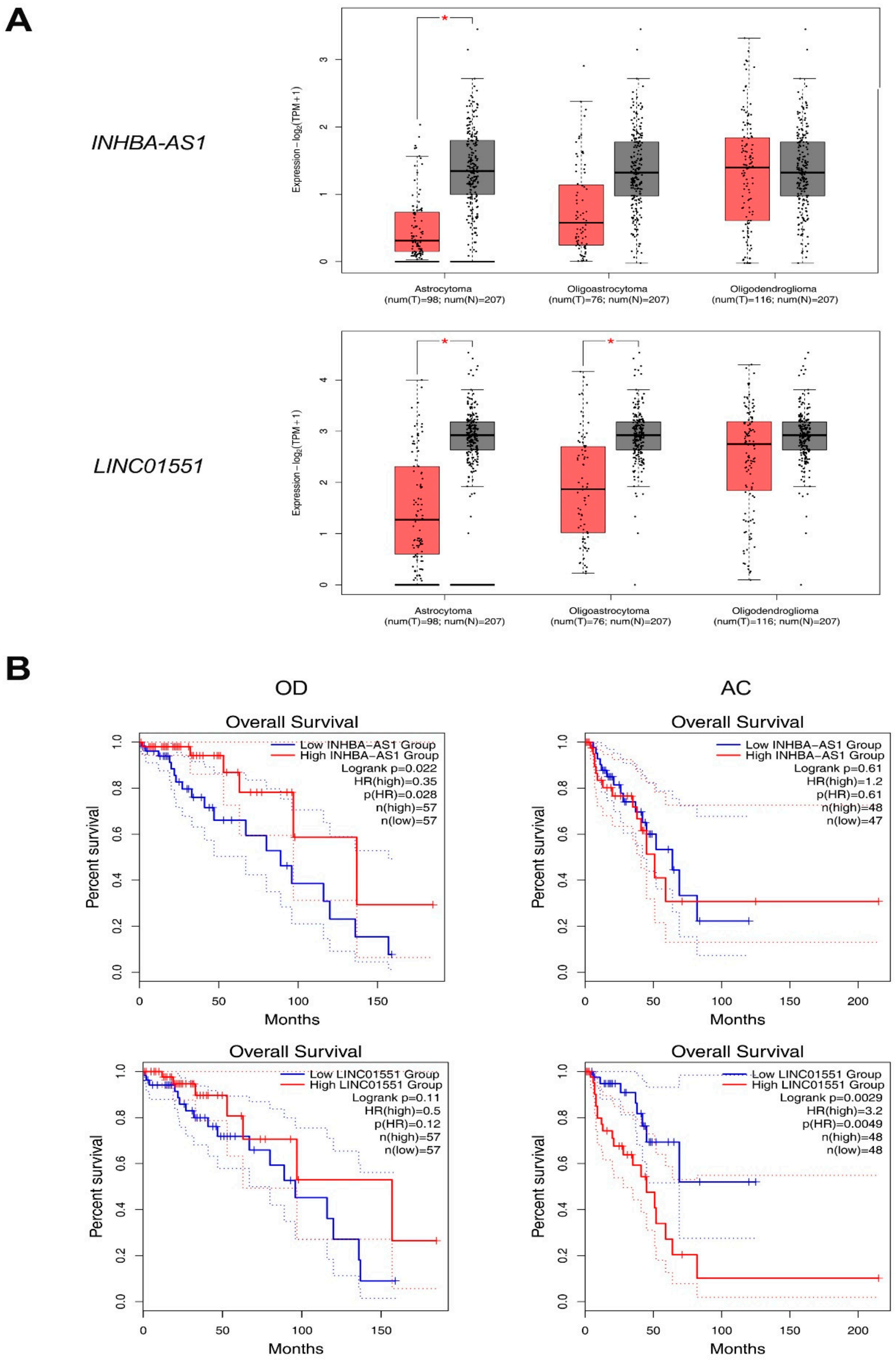

Figure S5. Compare to expression and survival rate on *INHBA-AS1, LINC01151* per glioma subtypes. Box plot for expression on *INHBA-AS1, LINC01151* among glioma subtypes (**A**). Red box indicates tumor expression from TCGA LGG database, grey box indicates normal tissue expression from GTEx

database. Kaplan Meier survival plot on *ABCC8, KCNJ11* in OD and AC patient in the TCGA database (**B**).

Table S1. ceRNA network evaluated by ARI using target coding genes. (k* = 2).

Source miRNA	No. Target Coding Gene	No. Target Non-Coding Gene	WGCNA Cluster	ARI	Network
hsa-miR-296-5p	78	6	Blue	0.921	Network01
hsa-miR-455-3p	68	1	Blue	0.896	Network02
hsa-miR-760	92	1	Blue	0.896	Network03
hsa-miR-1298-5p	43	4	Blue	0.896	Network04
hsa-miR-197-3p	78	5	Blue	0.872	Network05
hsa-miR-301a-5p	25	2	Blue	0.872	Network06
hsa-miR-1262	62	2	Blue	0.872	Network07
hsa-miR-186-5p	99	9	Blue	0.871	Network08
hsa-miR-301a-3p	83	2	Blue	0.847	Network09
hsa-miR-383-5p	97	1	Blue	0.824	Network10
hsa-miR-2114-3p	30	2	Blue	0.801	Network11
hsa-miR-204-5p	138	7	Blue	0.801	Network12
hsa-miR-7156-5p	31	6	Blue	0.800	Network13
hsa-miR-92b-3p	76	4	Blue	0.778	Network14
hsa-miR-3074-5p	31	9	Blue	0.778	Network15
hsa-miR-1298-3p	30	2	Blue	0.692	Network16
hsa-miR-3065-3p	37	3	Blue	0.466	-
hsa-miR-204-3p	61	13	Blue	0.372	-
hsa-miR-196b-5p	72	4	Blue	0.358	-
hsa-miR-3065-5p	18	3	Blue	0.329	-
hsa-miR-6715a-3p	22	1	Blue	0.328	-
hsa-miR-6507-5p	32	5	Blue	0.275	-
hsa-miR-3622a-3p	42	2	Blue	0.262	-
hsa-miR-216a-5p	89	6	Blue	0.225	-
hsa-miR-206	276	7	Turquoise	0.417	-
hsa-miR-888-5p	164	2	Turquoise	0.372	-

k*; Number of agglomerative cluster using for ARI.

Table S2. Gene list from ceRNA network.

Network	Source miRNA	Target Gene	Target Ensembl ID	Target Genetype
Network01	hsa-miR-296-5p	*AEN*	ENSG00000181026	Coding RNA
Network01	hsa-miR-296-5p	*AHSG*	ENSG00000145192	Coding RNA
Network01	hsa-miR-296-5p	*ARL4A*	ENSG00000122644	Coding RNA
Network01	hsa-miR-296-5p	*ATOH8*	ENSG00000168874	Coding RNA
Network01	hsa-miR-296-5p	*BLK*	ENSG00000136573	Coding RNA
Network01	hsa-miR-296-5p	*C1QTNF1*	ENSG00000173918	Coding RNA
Network01	hsa-miR-296-5p	*C20orf204*	ENSG00000196421	Coding RNA
Network01	hsa-miR-296-5p	*CACNG2*	ENSG00000166862	Coding RNA
Network01	hsa-miR-296-5p	*CACNG4*	ENSG00000075461	Coding RNA
Network01	hsa-miR-296-5p	*CAPN14*	ENSG00000214711	Coding RNA
Network01	hsa-miR-296-5p	*CCSER1*	ENSG00000184305	Coding RNA
Network01	hsa-miR-296-5p	*CDHR1*	ENSG00000148600	Coding RNA
Network01	hsa-miR-296-5p	*CDKN1A*	ENSG00000124762	Coding RNA
Network01	hsa-miR-296-5p	*CELSR3*	ENSG00000008300	Coding RNA
Network01	hsa-miR-296-5p	*CMBL*	ENSG00000164237	Coding RNA
Network01	hsa-miR-296-5p	*CMKLR1*	ENSG00000174600	Coding RNA
Network01	hsa-miR-296-5p	*CSPG5*	ENSG00000114646	Coding RNA
Network01	hsa-miR-296-5p	*DAND5*	ENSG00000179284	Coding RNA
Network01	hsa-miR-296-5p	*DHRS2*	ENSG00000100867	Coding RNA
Network01	hsa-miR-296-5p	*DRAM1*	ENSG00000136048	Coding RNA
Network01	hsa-miR-296-5p	*DRG2*	ENSG00000108591	Coding RNA
Network01	hsa-miR-296-5p	*DUSP4*	ENSG00000120875	Coding RNA
Network01	hsa-miR-296-5p	*DUSP9*	ENSG00000130829	Coding RNA
Network01	hsa-miR-296-5p	*EPN2*	ENSG00000072134	Coding RNA
Network01	hsa-miR-296-5p	*ETV4*	ENSG00000175832	Coding RNA
Network01	hsa-miR-296-5p	*FAM155B*	ENSG00000130054	Coding RNA
Network01	hsa-miR-296-5p	*FDXR*	ENSG00000161513	Coding RNA
Network01	hsa-miR-296-5p	*GCNT2*	ENSG00000111846	Coding RNA
Network01	hsa-miR-296-5p	*GFRA1*	ENSG00000151892	Coding RNA
Network01	hsa-miR-296-5p	*GLRA1*	ENSG00000145888	Coding RNA
Network01	hsa-miR-296-5p	*HCAR1*	ENSG00000196917	Coding RNA
Network01	hsa-miR-296-5p	*IL11RA*	ENSG00000137070	Coding RNA
Network01	hsa-miR-296-5p	*KCNG4*	ENSG00000168418	Coding RNA
Network01	hsa-miR-296-5p	*KCNIP2*	ENSG00000120049	Coding RNA
Network01	hsa-miR-296-5p	*KCNJ11*	ENSG00000187486	Coding RNA
Network01	hsa-miR-296-5p	*KCNK10*	ENSG00000100433	Coding RNA
Network01	hsa-miR-296-5p	*KCNK3*	ENSG00000171303	Coding RNA
Network01	hsa-miR-296-5p	*KREMEN2*	ENSG00000131650	Coding RNA
Network01	hsa-miR-296-5p	*LCN9*	ENSG00000148386	Coding RNA
Network01	hsa-miR-296-5p	*LPCAT1*	ENSG00000153395	Coding RNA
Network01	hsa-miR-296-5p	*MMP24*	ENSG00000125966	Coding RNA
Network01	hsa-miR-296-5p	*MYO3B*	ENSG00000071909	Coding RNA
Network01	hsa-miR-296-5p	*NGF*	ENSG00000134259	Coding RNA
Network01	hsa-miR-296-5p	*NGFR*	ENSG00000064300	Coding RNA
Network01	hsa-miR-296-5p	*NOG*	ENSG00000183691	Coding RNA
Network01	hsa-miR-296-5p	*P2RX3*	ENSG00000109991	Coding RNA
Network01	hsa-miR-296-5p	*PAX5*	ENSG00000196092	Coding RNA
Network01	hsa-miR-296-5p	*PDX1*	ENSG00000139515	Coding RNA
Network01	hsa-miR-296-5p	*PNPLA3*	ENSG00000100344	Coding RNA
Network01	hsa-miR-296-5p	*PTGFR*	ENSG00000122420	Coding RNA
Network01	hsa-miR-296-5p	*PTGIS*	ENSG00000124212	Coding RNA
Network01	hsa-miR-296-5p	*RAB31*	ENSG00000168461	Coding RNA

Network01	hsa-miR-296-5p	*RHBDL3*	ENSG00000141314	Coding RNA
Network01	hsa-miR-296-5p	*RORC*	ENSG00000143365	Coding RNA
Network01	hsa-miR-296-5p	*SALL4*	ENSG00000101115	Coding RNA
Network01	hsa-miR-296-5p	*SAPCD2*	ENSG00000186193	Coding RNA
Network01	hsa-miR-296-5p	*SCRT1*	ENSG00000261678	Coding RNA
Network01	hsa-miR-296-5p	*SEZ6*	ENSG00000063015	Coding RNA
Network01	hsa-miR-296-5p	*SLC16A10*	ENSG00000112394	Coding RNA
Network01	hsa-miR-296-5p	*SLC39A2*	ENSG00000165794	Coding RNA
Network01	hsa-miR-296-5p	*SLC5A2*	ENSG00000140675	Coding RNA
Network01	hsa-miR-296-5p	*SLC8A3*	ENSG00000100678	Coding RNA
Network01	hsa-miR-296-5p	*SLIT1*	ENSG00000187122	Coding RNA
Network01	hsa-miR-296-5p	*SPRY4*	ENSG00000187678	Coding RNA
Network01	hsa-miR-296-5p	*SRRM3*	ENSG00000177679	Coding RNA
Network01	hsa-miR-296-5p	*SUSD5*	ENSG00000173705	Coding RNA
Network01	hsa-miR-296-5p	*TBX5*	ENSG00000089225	Coding RNA
Network01	hsa-miR-296-5p	*TFAP2B*	ENSG00000008196	Coding RNA
Network01	hsa-miR-296-5p	*TLX1*	ENSG00000107807	Coding RNA
Network01	hsa-miR-296-5p	*TMEM158*	ENSG00000249992	Coding RNA
Network01	hsa-miR-296-5p	*TMEM97*	ENSG00000109084	Coding RNA
Network01	hsa-miR-296-5p	*TNFRSF10B*	ENSG00000120889	Coding RNA
Network01	hsa-miR-296-5p	*TOGARAM2*	ENSG00000189350	Coding RNA
Network01	hsa-miR-296-5p	*TRIM67*	ENSG00000119283	Coding RNA
Network01	hsa-miR-296-5p	*WSCD1*	ENSG00000179314	Coding RNA
Network01	hsa-miR-296-5p	*XKR7*	ENSG00000260903	Coding RNA
Network01	hsa-miR-296-5p	*XYLT1*	ENSG00000103489	Coding RNA
Network01	hsa-miR-296-5p	*ZFR2*	ENSG00000105278	Coding RNA
Network01	hsa-miR-296-5p	*LINC00648*	ENSG00000259129	lncRNA
Network01	hsa-miR-296-5p	*LINC01237*	ENSG00000233806	lncRNA
Network01	hsa-miR-296-5p	*LINC01460*	ENSG00000205334	lncRNA
Network01	hsa-miR-296-5p	*SMCR5*	ENSG00000226746	lncRNA
Network01	hsa-miR-296-5p	*TBX5-AS1*	ENSG00000255399	lncRNA
Network01	hsa-miR-296-5p	*TLX1NB*	ENSG00000236311	lncRNA
Network02	hsa-miR-455-3p	*ACAN*	ENSG00000157766	Coding RNA
Network02	hsa-miR-455-3p	*AEN*	ENSG00000181026	Coding RNA
Network02	hsa-miR-455-3p	*ANK1*	ENSG00000029534	Coding RNA
Network02	hsa-miR-455-3p	*ANO5*	ENSG00000171714	Coding RNA
Network02	hsa-miR-455-3p	*ARL4A*	ENSG00000122644	Coding RNA
Network02	hsa-miR-455-3p	*ATOH8*	ENSG00000168874	Coding RNA
Network02	hsa-miR-455-3p	*ATRX*	ENSG00000085224	Coding RNA
Network02	hsa-miR-455-3p	*BARHL1*	ENSG00000125492	Coding RNA
Network02	hsa-miR-455-3p	*BPIFB2*	ENSG00000078898	Coding RNA
Network02	hsa-miR-455-3p	*C20orf204*	ENSG00000196421	Coding RNA
Network02	hsa-miR-455-3p	*CACNG4*	ENSG00000075461	Coding RNA
Network02	hsa-miR-455-3p	*CAPN14*	ENSG00000214711	Coding RNA
Network02	hsa-miR-455-3p	*CDKN1A*	ENSG00000124762	Coding RNA
Network02	hsa-miR-455-3p	*CELSR3*	ENSG00000008300	Coding RNA
Network02	hsa-miR-455-3p	*CHGB*	ENSG00000089199	Coding RNA
Network02	hsa-miR-455-3p	*CMKLR1*	ENSG00000174600	Coding RNA
Network02	hsa-miR-455-3p	*CRISPLD2*	ENSG00000103196	Coding RNA
Network02	hsa-miR-455-3p	*DAND5*	ENSG00000179284	Coding RNA
Network02	hsa-miR-455-3p	*DUSP4*	ENSG00000120875	Coding RNA
Network02	hsa-miR-455-3p	*DUSP6*	ENSG00000139318	Coding RNA
Network02	hsa-miR-455-3p	*EDA2R*	ENSG00000131080	Coding RNA
Network02	hsa-miR-455-3p	*EPN2*	ENSG00000072134	Coding RNA
Network02	hsa-miR-455-3p	*ESRRG*	ENSG00000196482	Coding RNA
Network02	hsa-miR-455-3p	*FAM155B*	ENSG00000130054	Coding RNA

Network02	hsa-miR-455-3p	*FDXR*	ENSG00000161513	Coding RNA
Network02	hsa-miR-455-3p	*G6PC2*	ENSG00000152254	Coding RNA
Network02	hsa-miR-455-3p	*GCNT2*	ENSG00000111846	Coding RNA
Network02	hsa-miR-455-3p	*GJD2*	ENSG00000159248	Coding RNA
Network02	hsa-miR-455-3p	*IL11RA*	ENSG00000137070	Coding RNA
Network02	hsa-miR-455-3p	*KCNH7*	ENSG00000184611	Coding RNA
Network02	hsa-miR-455-3p	*KCNJ11*	ENSG00000187486	Coding RNA
Network02	hsa-miR-455-3p	*KCNK10*	ENSG00000100433	Coding RNA
Network02	hsa-miR-455-3p	*L1CAM*	ENSG00000198910	Coding RNA
Network02	hsa-miR-455-3p	*LPCAT1*	ENSG00000153395	Coding RNA
Network02	hsa-miR-455-3p	*LRP4*	ENSG00000134569	Coding RNA
Network02	hsa-miR-455-3p	*MB*	ENSG00000198125	Coding RNA
Network02	hsa-miR-455-3p	*MEGF11*	ENSG00000157890	Coding RNA
Network02	hsa-miR-455-3p	*MMP24*	ENSG00000125966	Coding RNA
Network02	hsa-miR-455-3p	*MYO3B*	ENSG00000071909	Coding RNA
Network02	hsa-miR-455-3p	*MYT1*	ENSG00000196132	Coding RNA
Network02	hsa-miR-455-3p	*NCR3LG1*	ENSG00000188211	Coding RNA
Network02	hsa-miR-455-3p	*NEUROD4*	ENSG00000123307	Coding RNA
Network02	hsa-miR-455-3p	*NGF*	ENSG00000134259	Coding RNA
Network02	hsa-miR-455-3p	*NGFR*	ENSG00000064300	Coding RNA
Network02	hsa-miR-455-3p	*PAX5*	ENSG00000196092	Coding RNA
Network02	hsa-miR-455-3p	*PTGFR*	ENSG00000122420	Coding RNA
Network02	hsa-miR-455-3p	*RAB31*	ENSG00000168461	Coding RNA
Network02	hsa-miR-455-3p	*SAPCD2*	ENSG00000186193	Coding RNA
Network02	hsa-miR-455-3p	*SCEL*	ENSG00000136155	Coding RNA
Network02	hsa-miR-455-3p	*SCML2*	ENSG00000102098	Coding RNA
Network02	hsa-miR-455-3p	*SCRT2*	ENSG00000215397	Coding RNA
Network02	hsa-miR-455-3p	*SEZ6L*	ENSG00000100095	Coding RNA
Network02	hsa-miR-455-3p	*SHISA9*	ENSG00000237515	Coding RNA
Network02	hsa-miR-455-3p	*SLAIN1*	ENSG00000139737	Coding RNA
Network02	hsa-miR-455-3p	*SLC35F1*	ENSG00000196376	Coding RNA
Network02	hsa-miR-455-3p	*SLC39A2*	ENSG00000165794	Coding RNA
Network02	hsa-miR-455-3p	*SPRY4*	ENSG00000187678	Coding RNA
Network02	hsa-miR-455-3p	*SRRM3*	ENSG00000177679	Coding RNA
Network02	hsa-miR-455-3p	*SUSD5*	ENSG00000173705	Coding RNA
Network02	hsa-miR-455-3p	*TERT*	ENSG00000164362	Coding RNA
Network02	hsa-miR-455-3p	*TMEM158*	ENSG00000249992	Coding RNA
Network02	hsa-miR-455-3p	*TNFRSF10B*	ENSG00000120889	Coding RNA
Network02	hsa-miR-455-3p	*TRPC3*	ENSG00000138741	Coding RNA
Network02	hsa-miR-455-3p	*UNC79*	ENSG00000133958	Coding RNA
Network02	hsa-miR-455-3p	*VASH2*	ENSG00000143494	Coding RNA
Network02	hsa-miR-455-3p	*XYLT1*	ENSG00000103489	Coding RNA
Network02	hsa-miR-455-3p	*ZBTB8B*	ENSG00000273274	Coding RNA
Network02	hsa-miR-455-3p	*ZDHHC22*	ENSG00000177108	Coding RNA
Network02	hsa-miR-455-3p	*LINC01586*	ENSG00000249487	lncRNA
Network03	hsa-miR-760	*ABCC8*	ENSG00000006071	Coding RNA
Network03	hsa-miR-760	*AEN*	ENSG00000181026	Coding RNA
Network03	hsa-miR-760	*AMZ1*	ENSG00000174945	Coding RNA
Network03	hsa-miR-760	*ANK1*	ENSG00000029534	Coding RNA
Network03	hsa-miR-760	*ARL4A*	ENSG00000122644	Coding RNA
Network03	hsa-miR-760	*ASCL5*	ENSG00000232237	Coding RNA
Network03	hsa-miR-760	*ATCAY*	ENSG00000167654	Coding RNA
Network03	hsa-miR-760	*ATOH8*	ENSG00000168874	Coding RNA
Network03	hsa-miR-760	*BLK*	ENSG00000136573	Coding RNA
Network03	hsa-miR-760	*C1QTNF1*	ENSG00000173918	Coding RNA
Network03	hsa-miR-760	*CACNG2*	ENSG00000166862	Coding RNA

Network03	hsa-miR-760	*CACNG4*	ENSG00000075461	Coding RNA
Network03	hsa-miR-760	*CAPN14*	ENSG00000214711	Coding RNA
Network03	hsa-miR-760	*CCSER1*	ENSG00000184305	Coding RNA
Network03	hsa-miR-760	*CDHR1*	ENSG00000148600	Coding RNA
Network03	hsa-miR-760	*CDKN1A*	ENSG00000124762	Coding RNA
Network03	hsa-miR-760	*CELSR3*	ENSG00000008300	Coding RNA
Network03	hsa-miR-760	*CMKLR1*	ENSG00000174600	Coding RNA
Network03	hsa-miR-760	*COL4A4*	ENSG00000081052	Coding RNA
Network03	hsa-miR-760	*CORO6*	ENSG00000167549	Coding RNA
Network03	hsa-miR-760	*CXCL13*	ENSG00000156234	Coding RNA
Network03	hsa-miR-760	*DAND5*	ENSG00000179284	Coding RNA
Network03	hsa-miR-760	*DGKG*	ENSG00000058866	Coding RNA
Network03	hsa-miR-760	*DRAM1*	ENSG00000136048	Coding RNA
Network03	hsa-miR-760	*DRG2*	ENSG00000108591	Coding RNA
Network03	hsa-miR-760	*DUSP4*	ENSG00000120875	Coding RNA
Network03	hsa-miR-760	*DUSP6*	ENSG00000139318	Coding RNA
Network03	hsa-miR-760	*EPN2*	ENSG00000072134	Coding RNA
Network03	hsa-miR-760	*ESRRG*	ENSG00000196482	Coding RNA
Network03	hsa-miR-760	*ETV5*	ENSG00000244405	Coding RNA
Network03	hsa-miR-760	*FAM155B*	ENSG00000130054	Coding RNA
Network03	hsa-miR-760	*FDXR*	ENSG00000161513	Coding RNA
Network03	hsa-miR-760	*FGFBP3*	ENSG00000174721	Coding RNA
Network03	hsa-miR-760	*G6PC2*	ENSG00000152254	Coding RNA
Network03	hsa-miR-760	*GCNT2*	ENSG00000111846	Coding RNA
Network03	hsa-miR-760	*GFRA1*	ENSG00000151892	Coding RNA
Network03	hsa-miR-760	*GRID2IP*	ENSG00000215045	Coding RNA
Network03	hsa-miR-760	*HCN4*	ENSG00000138622	Coding RNA
Network03	hsa-miR-760	*HIP1R*	ENSG00000130787	Coding RNA
Network03	hsa-miR-760	*IL11RA*	ENSG00000137070	Coding RNA
Network03	hsa-miR-760	*KCNIP1*	ENSG00000182132	Coding RNA
Network03	hsa-miR-760	*KCNIP2*	ENSG00000120049	Coding RNA
Network03	hsa-miR-760	*KLRC1*	ENSG00000134545	Coding RNA
Network03	hsa-miR-760	*L1CAM*	ENSG00000198910	Coding RNA
Network03	hsa-miR-760	*LINGO4*	ENSG00000213171	Coding RNA
Network03	hsa-miR-760	*LOR*	ENSG00000203782	Coding RNA
Network03	hsa-miR-760	*LPCAT1*	ENSG00000153395	Coding RNA
Network03	hsa-miR-760	*LRP4*	ENSG00000134569	Coding RNA
Network03	hsa-miR-760	*MLPH*	ENSG00000115648	Coding RNA
Network03	hsa-miR-760	*MMP24*	ENSG00000125966	Coding RNA
Network03	hsa-miR-760	*MYO18B*	ENSG00000133454	Coding RNA
Network03	hsa-miR-760	*MYO3B*	ENSG00000071909	Coding RNA
Network03	hsa-miR-760	*NAT16*	ENSG00000167011	Coding RNA
Network03	hsa-miR-760	*NCAN*	ENSG00000130287	Coding RNA
Network03	hsa-miR-760	*NCR3LG1*	ENSG00000188211	Coding RNA
Network03	hsa-miR-760	*NGFR*	ENSG00000064300	Coding RNA
Network03	hsa-miR-760	*NKX6-3*	ENSG00000165066	Coding RNA
Network03	hsa-miR-760	*PAX5*	ENSG00000196092	Coding RNA
Network03	hsa-miR-760	*PDX1*	ENSG00000139515	Coding RNA
Network03	hsa-miR-760	*PTGFR*	ENSG00000122420	Coding RNA
Network03	hsa-miR-760	*PTGIS*	ENSG00000124212	Coding RNA
Network03	hsa-miR-760	*RAB31*	ENSG00000168461	Coding RNA
Network03	hsa-miR-760	*RASSF2*	ENSG00000101265	Coding RNA
Network03	hsa-miR-760	*RHBDL3*	ENSG00000141314	Coding RNA
Network03	hsa-miR-760	*SAPCD2*	ENSG00000186193	Coding RNA
Network03	hsa-miR-760	*SCARA5*	ENSG00000168079	Coding RNA
Network03	hsa-miR-760	*SCEL*	ENSG00000136155	Coding RNA

Network03	hsa-miR-760	*SCML2*	ENSG00000102098	Coding RNA
Network03	hsa-miR-760	*SCRT1*	ENSG00000261678	Coding RNA
Network03	hsa-miR-760	*SCRT2*	ENSG00000215397	Coding RNA
Network03	hsa-miR-760	*SHC3*	ENSG00000148082	Coding RNA
Network03	hsa-miR-760	*SLAIN1*	ENSG00000139737	Coding RNA
Network03	hsa-miR-760	*SLC35F1*	ENSG00000196376	Coding RNA
Network03	hsa-miR-760	*SLC8A3*	ENSG00000100678	Coding RNA
Network03	hsa-miR-760	*SLIT1*	ENSG00000187122	Coding RNA
Network03	hsa-miR-760	*SPRY4*	ENSG00000187678	Coding RNA
Network03	hsa-miR-760	*SYCE2*	ENSG00000161860	Coding RNA
Network03	hsa-miR-760	*TFAP2B*	ENSG00000008196	Coding RNA
Network03	hsa-miR-760	*TLX1*	ENSG00000107807	Coding RNA
Network03	hsa-miR-760	*TMEM158*	ENSG00000249992	Coding RNA
Network03	hsa-miR-760	*TMEM97*	ENSG00000109084	Coding RNA
Network03	hsa-miR-760	*TNFRSF10B*	ENSG00000120889	Coding RNA
Network03	hsa-miR-760	*TNNT1*	ENSG00000105048	Coding RNA
Network03	hsa-miR-760	*TNR*	ENSG00000116147	Coding RNA
Network03	hsa-miR-760	*TRH*	ENSG00000170893	Coding RNA
Network03	hsa-miR-760	*TRIM67*	ENSG00000119283	Coding RNA
Network03	hsa-miR-760	*WSCD1*	ENSG00000179314	Coding RNA
Network03	hsa-miR-760	*XKR7*	ENSG00000260903	Coding RNA
Network03	hsa-miR-760	*XYLT1*	ENSG00000103489	Coding RNA
Network03	hsa-miR-760	*ZBTB8B*	ENSG00000273274	Coding RNA
Network03	hsa-miR-760	*ZDHHC22*	ENSG00000177108	Coding RNA
Network03	hsa-miR-760	*ZFR2*	ENSG00000105278	Coding RNA
Network03	hsa-miR-760	*LPAL2*	ENSG00000213071	Pseudogene
Network04	hsa-miR-1298-5p	*ACAN*	ENSG00000157766	Coding RNA
Network04	hsa-miR-1298-5p	*ACE*	ENSG00000159640	Coding RNA
Network04	hsa-miR-1298-5p	*AEN*	ENSG00000181026	Coding RNA
Network04	hsa-miR-1298-5p	*ANK1*	ENSG00000029534	Coding RNA
Network04	hsa-miR-1298-5p	*ARL4A*	ENSG00000122644	Coding RNA
Network04	hsa-miR-1298-5p	*ATOH8*	ENSG00000168874	Coding RNA
Network04	hsa-miR-1298-5p	*CACNG4*	ENSG00000075461	Coding RNA
Network04	hsa-miR-1298-5p	*CAPN14*	ENSG00000214711	Coding RNA
Network04	hsa-miR-1298-5p	*CD5L*	ENSG00000073754	Coding RNA
Network04	hsa-miR-1298-5p	*CDKN1A*	ENSG00000124762	Coding RNA
Network04	hsa-miR-1298-5p	*CMBL*	ENSG00000164237	Coding RNA
Network04	hsa-miR-1298-5p	*COL4A4*	ENSG00000081052	Coding RNA
Network04	hsa-miR-1298-5p	*DLX3*	ENSG00000064195	Coding RNA
Network04	hsa-miR-1298-5p	*DRAM1*	ENSG00000136048	Coding RNA
Network04	hsa-miR-1298-5p	*DUSP9*	ENSG00000130829	Coding RNA
Network04	hsa-miR-1298-5p	*EDA2R*	ENSG00000131080	Coding RNA
Network04	hsa-miR-1298-5p	*EPN2*	ENSG00000072134	Coding RNA
Network04	hsa-miR-1298-5p	*ETV5*	ENSG00000244405	Coding RNA
Network04	hsa-miR-1298-5p	*FAM155B*	ENSG00000130054	Coding RNA
Network04	hsa-miR-1298-5p	*GCNT2*	ENSG00000111846	Coding RNA
Network04	hsa-miR-1298-5p	*GFRA1*	ENSG00000151892	Coding RNA
Network04	hsa-miR-1298-5p	*HCRTR1*	ENSG00000121764	Coding RNA
Network04	hsa-miR-1298-5p	*KCNJ11*	ENSG00000187486	Coding RNA
Network04	hsa-miR-1298-5p	*KCNK3*	ENSG00000171303	Coding RNA
Network04	hsa-miR-1298-5p	*LPCAT1*	ENSG00000153395	Coding RNA
Network04	hsa-miR-1298-5p	*MICAL1*	ENSG00000135596	Coding RNA
Network04	hsa-miR-1298-5p	*MYO18B*	ENSG00000133454	Coding RNA
Network04	hsa-miR-1298-5p	*P2RX3*	ENSG00000109991	Coding RNA
Network04	hsa-miR-1298-5p	*PDX1*	ENSG00000139515	Coding RNA
Network04	hsa-miR-1298-5p	*PNPLA3*	ENSG00000100344	Coding RNA

Network04	hsa-miR-1298-5p	*RAB31*	ENSG00000168461	Coding RNA
Network04	hsa-miR-1298-5p	*SEZ6L*	ENSG00000100095	Coding RNA
Network04	hsa-miR-1298-5p	*SLAIN1*	ENSG00000139737	Coding RNA
Network04	hsa-miR-1298-5p	*SLC16A10*	ENSG00000112394	Coding RNA
Network04	hsa-miR-1298-5p	*SLC7A14*	ENSG00000013293	Coding RNA
Network04	hsa-miR-1298-5p	*SPRY4*	ENSG00000187678	Coding RNA
Network04	hsa-miR-1298-5p	*TFAP2B*	ENSG00000008196	Coding RNA
Network04	hsa-miR-1298-5p	*TRH*	ENSG00000170893	Coding RNA
Network04	hsa-miR-1298-5p	*TRMT9B*	ENSG00000250305	Coding RNA
Network04	hsa-miR-1298-5p	*TRPC3*	ENSG00000138741	Coding RNA
Network04	hsa-miR-1298-5p	*VASH2*	ENSG00000143494	Coding RNA
Network04	hsa-miR-1298-5p	*ZNF257*	ENSG00000197134	Coding RNA
Network04	hsa-miR-1298-5p	*ZNF676*	ENSG00000196109	Coding RNA
Network04	hsa-miR-1298-5p	*LINC01411*	ENSG00000249306	lncRNA
Network04	hsa-miR-1298-5p	*LINC01551*	ENSG00000186960	lncRNA
Network04	hsa-miR-1298-5p	*LINC01623*	ENSG00000225595	lncRNA
Network04	hsa-miR-1298-5p	*MROCKI*	ENSG00000227502	lncRNA
Network05	hsa-miR-197-3p	*ABCC8*	ENSG00000006071	Coding RNA
Network05	hsa-miR-197-3p	*AEN*	ENSG00000181026	Coding RNA
Network05	hsa-miR-197-3p	*AHSG*	ENSG00000145192	Coding RNA
Network05	hsa-miR-197-3p	*AMZ1*	ENSG00000174945	Coding RNA
Network05	hsa-miR-197-3p	*ASCL5*	ENSG00000232237	Coding RNA
Network05	hsa-miR-197-3p	*ATCAY*	ENSG00000167654	Coding RNA
Network05	hsa-miR-197-3p	*ATOH8*	ENSG00000168874	Coding RNA
Network05	hsa-miR-197-3p	*ATRX*	ENSG00000085224	Coding RNA
Network05	hsa-miR-197-3p	*C20orf204*	ENSG00000196421	Coding RNA
Network05	hsa-miR-197-3p	*CDHR1*	ENSG00000148600	Coding RNA
Network05	hsa-miR-197-3p	*CELSR3*	ENSG00000008300	Coding RNA
Network05	hsa-miR-197-3p	*CETP*	ENSG00000087237	Coding RNA
Network05	hsa-miR-197-3p	*CMBL*	ENSG00000164237	Coding RNA
Network05	hsa-miR-197-3p	*CMKLR1*	ENSG00000174600	Coding RNA
Network05	hsa-miR-197-3p	*COL4A3*	ENSG00000169031	Coding RNA
Network05	hsa-miR-197-3p	*COL9A1*	ENSG00000112280	Coding RNA
Network05	hsa-miR-197-3p	*CRISPLD2*	ENSG00000103196	Coding RNA
Network05	hsa-miR-197-3p	*CSPG5*	ENSG00000114646	Coding RNA
Network05	hsa-miR-197-3p	*DGKG*	ENSG00000058866	Coding RNA
Network05	hsa-miR-197-3p	*DLX3*	ENSG00000064195	Coding RNA
Network05	hsa-miR-197-3p	*DRAM1*	ENSG00000136048	Coding RNA
Network05	hsa-miR-197-3p	*DUSP4*	ENSG00000120875	Coding RNA
Network05	hsa-miR-197-3p	*DUSP6*	ENSG00000139318	Coding RNA
Network05	hsa-miR-197-3p	*EPN2*	ENSG00000072134	Coding RNA
Network05	hsa-miR-197-3p	*ETV4*	ENSG00000175832	Coding RNA
Network05	hsa-miR-197-3p	*ETV5*	ENSG00000244405	Coding RNA
Network05	hsa-miR-197-3p	*FAM155B*	ENSG00000130054	Coding RNA
Network05	hsa-miR-197-3p	*FDXR*	ENSG00000161513	Coding RNA
Network05	hsa-miR-197-3p	*GFRA1*	ENSG00000151892	Coding RNA
Network05	hsa-miR-197-3p	*GLRA1*	ENSG00000145888	Coding RNA
Network05	hsa-miR-197-3p	*GRID2IP*	ENSG00000215045	Coding RNA
Network05	hsa-miR-197-3p	*HCAR1*	ENSG00000196917	Coding RNA
Network05	hsa-miR-197-3p	*HCN4*	ENSG00000138622	Coding RNA
Network05	hsa-miR-197-3p	*HCRTR1*	ENSG00000121764	Coding RNA
Network05	hsa-miR-197-3p	*KCNIP2*	ENSG00000120049	Coding RNA
Network05	hsa-miR-197-3p	*KCNK10*	ENSG00000100433	Coding RNA
Network05	hsa-miR-197-3p	*KCNK3*	ENSG00000171303	Coding RNA
Network05	hsa-miR-197-3p	*KLRC1*	ENSG00000134545	Coding RNA
Network05	hsa-miR-197-3p	*KREMEN2*	ENSG00000131650	Coding RNA

Network05	hsa-miR-197-3p	*LPCAT1*	ENSG00000153395	Coding RNA
Network05	hsa-miR-197-3p	*LRP4*	ENSG00000134569	Coding RNA
Network05	hsa-miR-197-3p	*LRRC74A*	ENSG00000100565	Coding RNA
Network05	hsa-miR-197-3p	*MEGF11*	ENSG00000157890	Coding RNA
Network05	hsa-miR-197-3p	*MLPH*	ENSG00000115648	Coding RNA
Network05	hsa-miR-197-3p	*MMD2*	ENSG00000136297	Coding RNA
Network05	hsa-miR-197-3p	*NAT16*	ENSG00000167011	Coding RNA
Network05	hsa-miR-197-3p	*NCR3LG1*	ENSG00000188211	Coding RNA
Network05	hsa-miR-197-3p	*NEUROD4*	ENSG00000123307	Coding RNA
Network05	hsa-miR-197-3p	*NGFR*	ENSG00000064300	Coding RNA
Network05	hsa-miR-197-3p	*P2RX3*	ENSG00000109991	Coding RNA
Network05	hsa-miR-197-3p	*PAX5*	ENSG00000196092	Coding RNA
Network05	hsa-miR-197-3p	*PNPLA3*	ENSG00000100344	Coding RNA
Network05	hsa-miR-197-3p	*PRPH*	ENSG00000135406	Coding RNA
Network05	hsa-miR-197-3p	*PVRIG*	ENSG00000213413	Coding RNA
Network05	hsa-miR-197-3p	*RAB31*	ENSG00000168461	Coding RNA
Network05	hsa-miR-197-3p	*RASSF2*	ENSG00000101265	Coding RNA
Network05	hsa-miR-197-3p	*RHBDL3*	ENSG00000141314	Coding RNA
Network05	hsa-miR-197-3p	*SEZ6*	ENSG00000063015	Coding RNA
Network05	hsa-miR-197-3p	*SHC3*	ENSG00000148082	Coding RNA
Network05	hsa-miR-197-3p	*SHISA9*	ENSG00000237515	Coding RNA
Network05	hsa-miR-197-3p	*SLC16A10*	ENSG00000112394	Coding RNA
Network05	hsa-miR-197-3p	*SLC7A14*	ENSG00000013293	Coding RNA
Network05	hsa-miR-197-3p	*SLC8A3*	ENSG00000100678	Coding RNA
Network05	hsa-miR-197-3p	*SPRY4*	ENSG00000187678	Coding RNA
Network05	hsa-miR-197-3p	*SRRM3*	ENSG00000177679	Coding RNA
Network05	hsa-miR-197-3p	*ST6GAL2*	ENSG00000144057	Coding RNA
Network05	hsa-miR-197-3p	*STARD4*	ENSG00000164211	Coding RNA
Network05	hsa-miR-197-3p	*SUSD5*	ENSG00000173705	Coding RNA
Network05	hsa-miR-197-3p	*TBX5*	ENSG00000089225	Coding RNA
Network05	hsa-miR-197-3p	*TERT*	ENSG00000164362	Coding RNA
Network05	hsa-miR-197-3p	*TFAP2B*	ENSG00000008196	Coding RNA
Network05	hsa-miR-197-3p	*TNFRSF10B*	ENSG00000120889	Coding RNA
Network05	hsa-miR-197-3p	*TRMT9B*	ENSG00000250305	Coding RNA
Network05	hsa-miR-197-3p	*WSCD1*	ENSG00000179314	Coding RNA
Network05	hsa-miR-197-3p	*XYLT1*	ENSG00000103489	Coding RNA
Network05	hsa-miR-197-3p	*ZDHHC22*	ENSG00000177108	Coding RNA
Network05	hsa-miR-197-3p	*ZFR2*	ENSG00000105278	Coding RNA
Network05	hsa-miR-197-3p	*ZNF208*	ENSG00000160321	Coding RNA
Network05	hsa-miR-197-3p	*CD81-AS1*	ENSG00000238184	lncRNA
Network05	hsa-miR-197-3p	*FBXL19-AS1*	ENSG00000260852	lncRNA
Network05	hsa-miR-197-3p	*FZD10-AS1*	ENSG00000250208	lncRNA
Network05	hsa-miR-197-3p	*LINC02076*	ENSG00000220161	lncRNA
Network05	hsa-miR-197-3p	*SMCR5*	ENSG00000226746	lncRNA
Network06	hsa-miR-301a-5p	*ATRX*	ENSG00000085224	Coding RNA
Network06	hsa-miR-301a-5p	*BLK*	ENSG00000136573	Coding RNA
Network06	hsa-miR-301a-5p	*CAPN14*	ENSG00000214711	Coding RNA
Network06	hsa-miR-301a-5p	*CELSR3*	ENSG00000008300	Coding RNA
Network06	hsa-miR-301a-5p	*CMKLR1*	ENSG00000174600	Coding RNA
Network06	hsa-miR-301a-5p	*DUSP4*	ENSG00000120875	Coding RNA
Network06	hsa-miR-301a-5p	*DUSP6*	ENSG00000139318	Coding RNA
Network06	hsa-miR-301a-5p	*ESRRG*	ENSG00000196482	Coding RNA
Network06	hsa-miR-301a-5p	*FAM155B*	ENSG00000130054	Coding RNA
Network06	hsa-miR-301a-5p	*G6PC2*	ENSG00000152254	Coding RNA
Network06	hsa-miR-301a-5p	*INSYN2A*	ENSG00000188916	Coding RNA
Network06	hsa-miR-301a-5p	*KCNJ11*	ENSG00000187486	Coding RNA

Network06	hsa-miR-301a-5p	*NCAN*	ENSG00000130287	Coding RNA
Network06	hsa-miR-301a-5p	*NCR3LG1*	ENSG00000188211	Coding RNA
Network06	hsa-miR-301a-5p	*NGFR*	ENSG00000064300	Coding RNA
Network06	hsa-miR-301a-5p	*NOG*	ENSG00000183691	Coding RNA
Network06	hsa-miR-301a-5p	*PAX5*	ENSG00000196092	Coding RNA
Network06	hsa-miR-301a-5p	*PTGFR*	ENSG00000122420	Coding RNA
Network06	hsa-miR-301a-5p	*SCRT1*	ENSG00000261678	Coding RNA
Network06	hsa-miR-301a-5p	*SLAIN1*	ENSG00000139737	Coding RNA
Network06	hsa-miR-301a-5p	*SLC8A3*	ENSG00000100678	Coding RNA
Network06	hsa-miR-301a-5p	*SPRY4*	ENSG00000187678	Coding RNA
Network06	hsa-miR-301a-5p	*TLX1*	ENSG00000107807	Coding RNA
Network06	hsa-miR-301a-5p	*TNFRSF10B*	ENSG00000120889	Coding RNA
Network06	hsa-miR-301a-5p	*XKR7*	ENSG00000260903	Coding RNA
Network06	hsa-miR-301a-5p	*FZD10-AS1*	ENSG00000250208	lncRNA
Network06	hsa-miR-301a-5p	*SMCR5*	ENSG00000226746	lncRNA
Network07	hsa-miR-1262	*ACTC1*	ENSG00000159251	Coding RNA
Network07	hsa-miR-1262	*AEN*	ENSG00000181026	Coding RNA
Network07	hsa-miR-1262	*ARL4A*	ENSG00000122644	Coding RNA
Network07	hsa-miR-1262	*ATCAY*	ENSG00000167654	Coding RNA
Network07	hsa-miR-1262	*ATRX*	ENSG00000085224	Coding RNA
Network07	hsa-miR-1262	*CA10*	ENSG00000154975	Coding RNA
Network07	hsa-miR-1262	*CDKN1A*	ENSG00000124762	Coding RNA
Network07	hsa-miR-1262	*CELSR3*	ENSG00000008300	Coding RNA
Network07	hsa-miR-1262	*CMBL*	ENSG00000164237	Coding RNA
Network07	hsa-miR-1262	*CMKLR1*	ENSG00000174600	Coding RNA
Network07	hsa-miR-1262	*COL4A4*	ENSG00000081052	Coding RNA
Network07	hsa-miR-1262	*CRISPLD2*	ENSG00000103196	Coding RNA
Network07	hsa-miR-1262	*CSPG5*	ENSG00000114646	Coding RNA
Network07	hsa-miR-1262	*DAND5*	ENSG00000179284	Coding RNA
Network07	hsa-miR-1262	*DRAM1*	ENSG00000136048	Coding RNA
Network07	hsa-miR-1262	*DRG2*	ENSG00000108591	Coding RNA
Network07	hsa-miR-1262	*DUSP4*	ENSG00000120875	Coding RNA
Network07	hsa-miR-1262	*EDA2R*	ENSG00000131080	Coding RNA
Network07	hsa-miR-1262	*EPN2*	ENSG00000072134	Coding RNA
Network07	hsa-miR-1262	*FAM155B*	ENSG00000130054	Coding RNA
Network07	hsa-miR-1262	*FDXR*	ENSG00000161513	Coding RNA
Network07	hsa-miR-1262	*FOXF2*	ENSG00000137273	Coding RNA
Network07	hsa-miR-1262	*GCNT2*	ENSG00000111846	Coding RNA
Network07	hsa-miR-1262	*GFRA1*	ENSG00000151892	Coding RNA
Network07	hsa-miR-1262	*IL11RA*	ENSG00000137070	Coding RNA
Network07	hsa-miR-1262	*INSC*	ENSG00000188487	Coding RNA
Network07	hsa-miR-1262	*INSM2*	ENSG00000168348	Coding RNA
Network07	hsa-miR-1262	*INVS*	ENSG00000119509	Coding RNA
Network07	hsa-miR-1262	*KCNJ11*	ENSG00000187486	Coding RNA
Network07	hsa-miR-1262	*KCNK3*	ENSG00000171303	Coding RNA
Network07	hsa-miR-1262	*L1CAM*	ENSG00000198910	Coding RNA
Network07	hsa-miR-1262	*LPCAT1*	ENSG00000153395	Coding RNA
Network07	hsa-miR-1262	*MEGF11*	ENSG00000157890	Coding RNA
Network07	hsa-miR-1262	*MNX1*	ENSG00000130675	Coding RNA
Network07	hsa-miR-1262	*MYO3B*	ENSG00000071909	Coding RNA
Network07	hsa-miR-1262	*PAX5*	ENSG00000196092	Coding RNA
Network07	hsa-miR-1262	*RAB31*	ENSG00000168461	Coding RNA
Network07	hsa-miR-1262	*RASSF2*	ENSG00000101265	Coding RNA
Network07	hsa-miR-1262	*RPL22L1*	ENSG00000163584	Coding RNA
Network07	hsa-miR-1262	*RUFY4*	ENSG00000188282	Coding RNA
Network07	hsa-miR-1262	*SCEL*	ENSG00000136155	Coding RNA

Network07	hsa-miR-1262	*SCML2*	ENSG00000102098	Coding RNA
Network07	hsa-miR-1262	*SCRT2*	ENSG00000215397	Coding RNA
Network07	hsa-miR-1262	*SEZ6L*	ENSG00000100095	Coding RNA
Network07	hsa-miR-1262	*SLAIN1*	ENSG00000139737	Coding RNA
Network07	hsa-miR-1262	*SLC16A10*	ENSG00000112394	Coding RNA
Network07	hsa-miR-1262	*SLC8A3*	ENSG00000100678	Coding RNA
Network07	hsa-miR-1262	*SPRY4*	ENSG00000187678	Coding RNA
Network07	hsa-miR-1262	*STARD4*	ENSG00000164211	Coding RNA
Network07	hsa-miR-1262	*SYCE1*	ENSG00000171772	Coding RNA
Network07	hsa-miR-1262	*SYCE2*	ENSG00000161860	Coding RNA
Network07	hsa-miR-1262	*TBX5*	ENSG00000089225	Coding RNA
Network07	hsa-miR-1262	*TERT*	ENSG00000164362	Coding RNA
Network07	hsa-miR-1262	*TMEM158*	ENSG00000249992	Coding RNA
Network07	hsa-miR-1262	*TMEM97*	ENSG00000109084	Coding RNA
Network07	hsa-miR-1262	*TNFRSF10B*	ENSG00000120889	Coding RNA
Network07	hsa-miR-1262	*TRIM67*	ENSG00000119283	Coding RNA
Network07	hsa-miR-1262	*TRPC3*	ENSG00000138741	Coding RNA
Network07	hsa-miR-1262	*VASH2*	ENSG00000143494	Coding RNA
Network07	hsa-miR-1262	*ZDHHC22*	ENSG00000177108	Coding RNA
Network07	hsa-miR-1262	*ZNF257*	ENSG00000197134	Coding RNA
Network07	hsa-miR-1262	*ZNF676*	ENSG00000196109	Coding RNA
Network07	hsa-miR-1262	*CISTR*	ENSG00000260492	lncRNA
Network07	hsa-miR-1262	*LINC01132*	ENSG00000227630	lncRNA
Network08	hsa-miR-186-5p	*ACAT2*	ENSG00000120437	Coding RNA
Network08	hsa-miR-186-5p	*ACTC1*	ENSG00000159251	Coding RNA
Network08	hsa-miR-186-5p	*AEN*	ENSG00000181026	Coding RNA
Network08	hsa-miR-186-5p	*ALK*	ENSG00000171094	Coding RNA
Network08	hsa-miR-186-5p	*AMZ1*	ENSG00000174945	Coding RNA
Network08	hsa-miR-186-5p	*ANKRD55*	ENSG00000164512	Coding RNA
Network08	hsa-miR-186-5p	*ANO5*	ENSG00000171714	Coding RNA
Network08	hsa-miR-186-5p	*ATRX*	ENSG00000085224	Coding RNA
Network08	hsa-miR-186-5p	*C1QTNF1*	ENSG00000173918	Coding RNA
Network08	hsa-miR-186-5p	*CA10*	ENSG00000154975	Coding RNA
Network08	hsa-miR-186-5p	*CACNG2*	ENSG00000166862	Coding RNA
Network08	hsa-miR-186-5p	*CACNG4*	ENSG00000075461	Coding RNA
Network08	hsa-miR-186-5p	*CCSER1*	ENSG00000184305	Coding RNA
Network08	hsa-miR-186-5p	*CD5L*	ENSG00000073754	Coding RNA
Network08	hsa-miR-186-5p	*CDKN1A*	ENSG00000124762	Coding RNA
Network08	hsa-miR-186-5p	*CELSR3*	ENSG00000008300	Coding RNA
Network08	hsa-miR-186-5p	*CHGB*	ENSG00000089199	Coding RNA
Network08	hsa-miR-186-5p	*CMKLR1*	ENSG00000174600	Coding RNA
Network08	hsa-miR-186-5p	*COL17A1*	ENSG00000065618	Coding RNA
Network08	hsa-miR-186-5p	*COL4A3*	ENSG00000169031	Coding RNA
Network08	hsa-miR-186-5p	*COL4A4*	ENSG00000081052	Coding RNA
Network08	hsa-miR-186-5p	*CREB3L1*	ENSG00000157613	Coding RNA
Network08	hsa-miR-186-5p	*CREB3L4*	ENSG00000143578	Coding RNA
Network08	hsa-miR-186-5p	*CRISPLD2*	ENSG00000103196	Coding RNA
Network08	hsa-miR-186-5p	*CSPG5*	ENSG00000114646	Coding RNA
Network08	hsa-miR-186-5p	*CXCL13*	ENSG00000156234	Coding RNA
Network08	hsa-miR-186-5p	*DGKG*	ENSG00000058866	Coding RNA
Network08	hsa-miR-186-5p	*DGKK*	ENSG00000274588	Coding RNA
Network08	hsa-miR-186-5p	*DNAH14*	ENSG00000185842	Coding RNA
Network08	hsa-miR-186-5p	*DRAM1*	ENSG00000136048	Coding RNA
Network08	hsa-miR-186-5p	*DUSP6*	ENSG00000139318	Coding RNA
Network08	hsa-miR-186-5p	*DUSP9*	ENSG00000130829	Coding RNA
Network08	hsa-miR-186-5p	*EDA2R*	ENSG00000131080	Coding RNA

Network08	hsa-miR-186-5p	*EPN2*	ENSG00000072134	Coding RNA
Network08	hsa-miR-186-5p	*ESRRG*	ENSG00000196482	Coding RNA
Network08	hsa-miR-186-5p	*ETV4*	ENSG00000175832	Coding RNA
Network08	hsa-miR-186-5p	*ETV5*	ENSG00000244405	Coding RNA
Network08	hsa-miR-186-5p	*FGFBP3*	ENSG00000174721	Coding RNA
Network08	hsa-miR-186-5p	*FOXF2*	ENSG00000137273	Coding RNA
Network08	hsa-miR-186-5p	*GALR1*	ENSG00000166573	Coding RNA
Network08	hsa-miR-186-5p	*GCNT2*	ENSG00000111846	Coding RNA
Network08	hsa-miR-186-5p	*GFRA1*	ENSG00000151892	Coding RNA
Network08	hsa-miR-186-5p	*HCN4*	ENSG00000138622	Coding RNA
Network08	hsa-miR-186-5p	*HIP1R*	ENSG00000130787	Coding RNA
Network08	hsa-miR-186-5p	*INSC*	ENSG00000188487	Coding RNA
Network08	hsa-miR-186-5p	*INSYN2A*	ENSG00000188916	Coding RNA
Network08	hsa-miR-186-5p	*INVS*	ENSG00000119509	Coding RNA
Network08	hsa-miR-186-5p	*KCNH7*	ENSG00000184611	Coding RNA
Network08	hsa-miR-186-5p	*KCNK10*	ENSG00000100433	Coding RNA
Network08	hsa-miR-186-5p	*LHFPL3*	ENSG00000187416	Coding RNA
Network08	hsa-miR-186-5p	*LPCAT1*	ENSG00000153395	Coding RNA
Network08	hsa-miR-186-5p	*LRP4*	ENSG00000134569	Coding RNA
Network08	hsa-miR-186-5p	*MEGF11*	ENSG00000157890	Coding RNA
Network08	hsa-miR-186-5p	*MLPH*	ENSG00000115648	Coding RNA
Network08	hsa-miR-186-5p	*MNX1*	ENSG00000130675	Coding RNA
Network08	hsa-miR-186-5p	*MYO3B*	ENSG00000071909	Coding RNA
Network08	hsa-miR-186-5p	*MYT1*	ENSG00000196132	Coding RNA
Network08	hsa-miR-186-5p	*NAT16*	ENSG00000167011	Coding RNA
Network08	hsa-miR-186-5p	*NCAN*	ENSG00000130287	Coding RNA
Network08	hsa-miR-186-5p	*NCR3LG1*	ENSG00000188211	Coding RNA
Network08	hsa-miR-186-5p	*NEUROD4*	ENSG00000123307	Coding RNA
Network08	hsa-miR-186-5p	*NGF*	ENSG00000134259	Coding RNA
Network08	hsa-miR-186-5p	*NGFR*	ENSG00000064300	Coding RNA
Network08	hsa-miR-186-5p	*NOG*	ENSG00000183691	Coding RNA
Network08	hsa-miR-186-5p	*PAX5*	ENSG00000196092	Coding RNA
Network08	hsa-miR-186-5p	*PNPLA3*	ENSG00000100344	Coding RNA
Network08	hsa-miR-186-5p	*PTGFR*	ENSG00000122420	Coding RNA
Network08	hsa-miR-186-5p	*PTGIS*	ENSG00000124212	Coding RNA
Network08	hsa-miR-186-5p	*RAB31*	ENSG00000168461	Coding RNA
Network08	hsa-miR-186-5p	*RASSF2*	ENSG00000101265	Coding RNA
Network08	hsa-miR-186-5p	*RHBDL3*	ENSG00000141314	Coding RNA
Network08	hsa-miR-186-5p	*RORC*	ENSG00000143365	Coding RNA
Network08	hsa-miR-186-5p	*SALL4*	ENSG00000101115	Coding RNA
Network08	hsa-miR-186-5p	*SCEL*	ENSG00000136155	Coding RNA
Network08	hsa-miR-186-5p	*SCML2*	ENSG00000102098	Coding RNA
Network08	hsa-miR-186-5p	*SEZ6L*	ENSG00000100095	Coding RNA
Network08	hsa-miR-186-5p	*SHC3*	ENSG00000148082	Coding RNA
Network08	hsa-miR-186-5p	*SHISA9*	ENSG00000237515	Coding RNA
Network08	hsa-miR-186-5p	*SLAIN1*	ENSG00000139737	Coding RNA
Network08	hsa-miR-186-5p	*SLC16A10*	ENSG00000112394	Coding RNA
Network08	hsa-miR-186-5p	*SLC17A8*	ENSG00000179520	Coding RNA
Network08	hsa-miR-186-5p	*SLC35F1*	ENSG00000196376	Coding RNA
Network08	hsa-miR-186-5p	*SLC8A3*	ENSG00000100678	Coding RNA
Network08	hsa-miR-186-5p	*SPRY4*	ENSG00000187678	Coding RNA
Network08	hsa-miR-186-5p	*ST6GAL2*	ENSG00000144057	Coding RNA
Network08	hsa-miR-186-5p	*STARD4*	ENSG00000164211	Coding RNA
Network08	hsa-miR-186-5p	*TBX5*	ENSG00000089225	Coding RNA
Network08	hsa-miR-186-5p	*TFAP2B*	ENSG00000008196	Coding RNA
Network08	hsa-miR-186-5p	*TMEM26*	ENSG00000196932	Coding RNA

Network08	hsa-miR-186-5p	*TNFRSF10B*	ENSG00000120889	Coding RNA
Network08	hsa-miR-186-5p	*TRIM67*	ENSG00000119283	Coding RNA
Network08	hsa-miR-186-5p	*TRMT9B*	ENSG00000250305	Coding RNA
Network08	hsa-miR-186-5p	*UNC79*	ENSG00000133958	Coding RNA
Network08	hsa-miR-186-5p	*VASH2*	ENSG00000143494	Coding RNA
Network08	hsa-miR-186-5p	*VWC2L*	ENSG00000174453	Coding RNA
Network08	hsa-miR-186-5p	*XYLT1*	ENSG00000103489	Coding RNA
Network08	hsa-miR-186-5p	*ZBTB8B*	ENSG00000273274	Coding RNA
Network08	hsa-miR-186-5p	*ZDHHC22*	ENSG00000177108	Coding RNA
Network08	hsa-miR-186-5p	*ZFR2*	ENSG00000105278	Coding RNA
Network08	hsa-miR-186-5p	*FZD10-AS1*	ENSG00000250208	lncRNA
Network08	hsa-miR-186-5p	*INHBA-AS1*	ENSG00000224116	lncRNA
Network08	hsa-miR-186-5p	*LINC00648*	ENSG00000259129	lncRNA
Network08	hsa-miR-186-5p	*LINC01132*	ENSG00000227630	lncRNA
Network08	hsa-miR-186-5p	*LINC01237*	ENSG00000233806	lncRNA
Network08	hsa-miR-186-5p	*LINC01460*	ENSG00000205334	lncRNA
Network08	hsa-miR-186-5p	*TM4SF19-AS1*	ENSG00000235897	lncRNA
Network08	hsa-miR-186-5p	*LPAL2*	ENSG00000213071	Pseudogene
Network08	hsa-miR-186-5p	*ZNF876P*	ENSG00000198155	Pseudogene
Network09	hsa-miR-301a-3p	*ACAT2*	ENSG00000120437	Coding RNA
Network09	hsa-miR-301a-3p	*ACTC1*	ENSG00000159251	Coding RNA
Network09	hsa-miR-301a-3p	*AEN*	ENSG00000181026	Coding RNA
Network09	hsa-miR-301a-3p	*AHSG*	ENSG00000145192	Coding RNA
Network09	hsa-miR-301a-3p	*AMZ1*	ENSG00000174945	Coding RNA
Network09	hsa-miR-301a-3p	*ANKRD55*	ENSG00000164512	Coding RNA
Network09	hsa-miR-301a-3p	*ANO5*	ENSG00000171714	Coding RNA
Network09	hsa-miR-301a-3p	*ARL4A*	ENSG00000122644	Coding RNA
Network09	hsa-miR-301a-3p	*ATOH8*	ENSG00000168874	Coding RNA
Network09	hsa-miR-301a-3p	*ATRX*	ENSG00000085224	Coding RNA
Network09	hsa-miR-301a-3p	*BLK*	ENSG00000136573	Coding RNA
Network09	hsa-miR-301a-3p	*C20orf204*	ENSG00000196421	Coding RNA
Network09	hsa-miR-301a-3p	*CA10*	ENSG00000154975	Coding RNA
Network09	hsa-miR-301a-3p	*CCSER1*	ENSG00000184305	Coding RNA
Network09	hsa-miR-301a-3p	*CD5L*	ENSG00000073754	Coding RNA
Network09	hsa-miR-301a-3p	*CDKN1A*	ENSG00000124762	Coding RNA
Network09	hsa-miR-301a-3p	*CHRDL2*	ENSG00000054938	Coding RNA
Network09	hsa-miR-301a-3p	*CMBL*	ENSG00000164237	Coding RNA
Network09	hsa-miR-301a-3p	*CMKLR1*	ENSG00000174600	Coding RNA
Network09	hsa-miR-301a-3p	*COL4A3*	ENSG00000169031	Coding RNA
Network09	hsa-miR-301a-3p	*COL4A4*	ENSG00000081052	Coding RNA
Network09	hsa-miR-301a-3p	*CSPG5*	ENSG00000114646	Coding RNA
Network09	hsa-miR-301a-3p	*CXCL13*	ENSG00000156234	Coding RNA
Network09	hsa-miR-301a-3p	*DGKG*	ENSG00000058866	Coding RNA
Network09	hsa-miR-301a-3p	*DNAH14*	ENSG00000185842	Coding RNA
Network09	hsa-miR-301a-3p	*DRG2*	ENSG00000108591	Coding RNA
Network09	hsa-miR-301a-3p	*DUSP9*	ENSG00000130829	Coding RNA
Network09	hsa-miR-301a-3p	*EDA2R*	ENSG00000131080	Coding RNA
Network09	hsa-miR-301a-3p	*EPN2*	ENSG00000072134	Coding RNA
Network09	hsa-miR-301a-3p	*ESRRG*	ENSG00000196482	Coding RNA
Network09	hsa-miR-301a-3p	*ETV5*	ENSG00000244405	Coding RNA
Network09	hsa-miR-301a-3p	*FAM155B*	ENSG00000130054	Coding RNA
Network09	hsa-miR-301a-3p	*FOXF2*	ENSG00000137273	Coding RNA
Network09	hsa-miR-301a-3p	*GCNT2*	ENSG00000111846	Coding RNA
Network09	hsa-miR-301a-3p	*GFRA1*	ENSG00000151892	Coding RNA
Network09	hsa-miR-301a-3p	*HIP1R*	ENSG00000130787	Coding RNA
Network09	hsa-miR-301a-3p	*IL11RA*	ENSG00000137070	Coding RNA

Network09	hsa-miR-301a-3p	*INSM2*	ENSG00000168348	Coding RNA
Network09	hsa-miR-301a-3p	*KCNIP2*	ENSG00000120049	Coding RNA
Network09	hsa-miR-301a-3p	*KCNJ11*	ENSG00000187486	Coding RNA
Network09	hsa-miR-301a-3p	*KCNK10*	ENSG00000100433	Coding RNA
Network09	hsa-miR-301a-3p	*LPCAT1*	ENSG00000153395	Coding RNA
Network09	hsa-miR-301a-3p	*LRP4*	ENSG00000134569	Coding RNA
Network09	hsa-miR-301a-3p	*MLPH*	ENSG00000115648	Coding RNA
Network09	hsa-miR-301a-3p	*MNX1*	ENSG00000130675	Coding RNA
Network09	hsa-miR-301a-3p	*MYO3B*	ENSG00000071909	Coding RNA
Network09	hsa-miR-301a-3p	*MYT1*	ENSG00000196132	Coding RNA
Network09	hsa-miR-301a-3p	*NMU*	ENSG00000109255	Coding RNA
Network09	hsa-miR-301a-3p	*NOG*	ENSG00000183691	Coding RNA
Network09	hsa-miR-301a-3p	*NOX4*	ENSG00000086991	Coding RNA
Network09	hsa-miR-301a-3p	*PDX1*	ENSG00000139515	Coding RNA
Network09	hsa-miR-301a-3p	*PTGFR*	ENSG00000122420	Coding RNA
Network09	hsa-miR-301a-3p	*RAB31*	ENSG00000168461	Coding RNA
Network09	hsa-miR-301a-3p	*RASSF2*	ENSG00000101265	Coding RNA
Network09	hsa-miR-301a-3p	*RORC*	ENSG00000143365	Coding RNA
Network09	hsa-miR-301a-3p	*SCARA5*	ENSG00000168079	Coding RNA
Network09	hsa-miR-301a-3p	*SCML2*	ENSG00000102098	Coding RNA
Network09	hsa-miR-301a-3p	*SCRT1*	ENSG00000261678	Coding RNA
Network09	hsa-miR-301a-3p	*SHC3*	ENSG00000148082	Coding RNA
Network09	hsa-miR-301a-3p	*SHISA9*	ENSG00000237515	Coding RNA
Network09	hsa-miR-301a-3p	*SLAIN1*	ENSG00000139737	Coding RNA
Network09	hsa-miR-301a-3p	*SLC16A10*	ENSG00000112394	Coding RNA
Network09	hsa-miR-301a-3p	*SLC35F1*	ENSG00000196376	Coding RNA
Network09	hsa-miR-301a-3p	*SLC7A14*	ENSG00000013293	Coding RNA
Network09	hsa-miR-301a-3p	*SPRY4*	ENSG00000187678	Coding RNA
Network09	hsa-miR-301a-3p	*ST6GAL2*	ENSG00000144057	Coding RNA
Network09	hsa-miR-301a-3p	*SUSD5*	ENSG00000173705	Coding RNA
Network09	hsa-miR-301a-3p	*TBX5*	ENSG00000089225	Coding RNA
Network09	hsa-miR-301a-3p	*TFAP2B*	ENSG00000008196	Coding RNA
Network09	hsa-miR-301a-3p	*TLX1*	ENSG00000107807	Coding RNA
Network09	hsa-miR-301a-3p	*TMEM158*	ENSG00000249992	Coding RNA
Network09	hsa-miR-301a-3p	*TMEM26*	ENSG00000196932	Coding RNA
Network09	hsa-miR-301a-3p	*TMEM97*	ENSG00000109084	Coding RNA
Network09	hsa-miR-301a-3p	*TNFRSF10B*	ENSG00000120889	Coding RNA
Network09	hsa-miR-301a-3p	*TRIM67*	ENSG00000119283	Coding RNA
Network09	hsa-miR-301a-3p	*TRMT9B*	ENSG00000250305	Coding RNA
Network09	hsa-miR-301a-3p	*TRPC3*	ENSG00000138741	Coding RNA
Network09	hsa-miR-301a-3p	*UNC79*	ENSG00000133958	Coding RNA
Network09	hsa-miR-301a-3p	*VASH2*	ENSG00000143494	Coding RNA
Network09	hsa-miR-301a-3p	*ZDHHC22*	ENSG00000177108	Coding RNA
Network09	hsa-miR-301a-3p	*ZFR2*	ENSG00000105278	Coding RNA
Network09	hsa-miR-301a-3p	*ZNF208*	ENSG00000160321	Coding RNA
Network09	hsa-miR-301a-3p	*ZNF257*	ENSG00000197134	Coding RNA
Network09	hsa-miR-301a-3p	*LINC00574*	ENSG00000231690	lncRNA
Network09	hsa-miR-301a-3p	*LINC01551*	ENSG00000186960	lncRNA
Network10	hsa-miR-383-5p	*ACE*	ENSG00000159640	Coding RNA
Network10	hsa-miR-383-5p	*AEN*	ENSG00000181026	Coding RNA
Network10	hsa-miR-383-5p	*ALK*	ENSG00000171094	Coding RNA
Network10	hsa-miR-383-5p	*ANK1*	ENSG00000029534	Coding RNA
Network10	hsa-miR-383-5p	*ANO5*	ENSG00000171714	Coding RNA
Network10	hsa-miR-383-5p	*ARL4A*	ENSG00000122644	Coding RNA
Network10	hsa-miR-383-5p	*ASIC4*	ENSG00000072182	Coding RNA
Network10	hsa-miR-383-5p	*ATCAY*	ENSG00000167654	Coding RNA

Network10	hsa-miR-383-5p	*ATOH8*	ENSG00000168874	Coding RNA
Network10	hsa-miR-383-5p	*ATRX*	ENSG00000085224	Coding RNA
Network10	hsa-miR-383-5p	*BARHL1*	ENSG00000125492	Coding RNA
Network10	hsa-miR-383-5p	*BLK*	ENSG00000136573	Coding RNA
Network10	hsa-miR-383-5p	*C1QTNF1*	ENSG00000173918	Coding RNA
Network10	hsa-miR-383-5p	*CACNG2*	ENSG00000166862	Coding RNA
Network10	hsa-miR-383-5p	*CAPN11*	ENSG00000137225	Coding RNA
Network10	hsa-miR-383-5p	*CCSER1*	ENSG00000184305	Coding RNA
Network10	hsa-miR-383-5p	*CD5L*	ENSG00000073754	Coding RNA
Network10	hsa-miR-383-5p	*CDHR1*	ENSG00000148600	Coding RNA
Network10	hsa-miR-383-5p	*CDKN1A*	ENSG00000124762	Coding RNA
Network10	hsa-miR-383-5p	*CELSR3*	ENSG00000008300	Coding RNA
Network10	hsa-miR-383-5p	*CLVS1*	ENSG00000177182	Coding RNA
Network10	hsa-miR-383-5p	*COL17A1*	ENSG00000065618	Coding RNA
Network10	hsa-miR-383-5p	*COL4A3*	ENSG00000169031	Coding RNA
Network10	hsa-miR-383-5p	*COL4A4*	ENSG00000081052	Coding RNA
Network10	hsa-miR-383-5p	*CPA5*	ENSG00000158525	Coding RNA
Network10	hsa-miR-383-5p	*CREB3L4*	ENSG00000143578	Coding RNA
Network10	hsa-miR-383-5p	*CSPG5*	ENSG00000114646	Coding RNA
Network10	hsa-miR-383-5p	*DHRS2*	ENSG00000100867	Coding RNA
Network10	hsa-miR-383-5p	*DNAH14*	ENSG00000185842	Coding RNA
Network10	hsa-miR-383-5p	*DNASE1L3*	ENSG00000163687	Coding RNA
Network10	hsa-miR-383-5p	*DRAM1*	ENSG00000136048	Coding RNA
Network10	hsa-miR-383-5p	*DUSP4*	ENSG00000120875	Coding RNA
Network10	hsa-miR-383-5p	*DUSP6*	ENSG00000139318	Coding RNA
Network10	hsa-miR-383-5p	*EDA2R*	ENSG00000131080	Coding RNA
Network10	hsa-miR-383-5p	*EPN2*	ENSG00000072134	Coding RNA
Network10	hsa-miR-383-5p	*ESRRG*	ENSG00000196482	Coding RNA
Network10	hsa-miR-383-5p	*ETV5*	ENSG00000244405	Coding RNA
Network10	hsa-miR-383-5p	*FAM155B*	ENSG00000130054	Coding RNA
Network10	hsa-miR-383-5p	*FDXR*	ENSG00000161513	Coding RNA
Network10	hsa-miR-383-5p	*FGFBP3*	ENSG00000174721	Coding RNA
Network10	hsa-miR-383-5p	*GCNT2*	ENSG00000111846	Coding RNA
Network10	hsa-miR-383-5p	*GFRA1*	ENSG00000151892	Coding RNA
Network10	hsa-miR-383-5p	*GRID2IP*	ENSG00000215045	Coding RNA
Network10	hsa-miR-383-5p	*HCAR1*	ENSG00000196917	Coding RNA
Network10	hsa-miR-383-5p	*HIP1R*	ENSG00000130787	Coding RNA
Network10	hsa-miR-383-5p	*HS3ST3A1*	ENSG00000153976	Coding RNA
Network10	hsa-miR-383-5p	*IL11RA*	ENSG00000137070	Coding RNA
Network10	hsa-miR-383-5p	*KCNH7*	ENSG00000184611	Coding RNA
Network10	hsa-miR-383-5p	*KCNIP1*	ENSG00000182132	Coding RNA
Network10	hsa-miR-383-5p	*KCNJ11*	ENSG00000187486	Coding RNA
Network10	hsa-miR-383-5p	*KCNK10*	ENSG00000100433	Coding RNA
Network10	hsa-miR-383-5p	*L1CAM*	ENSG00000198910	Coding RNA
Network10	hsa-miR-383-5p	*LHFPL3*	ENSG00000187416	Coding RNA
Network10	hsa-miR-383-5p	*LPCAT1*	ENSG00000153395	Coding RNA
Network10	hsa-miR-383-5p	*LRP4*	ENSG00000134569	Coding RNA
Network10	hsa-miR-383-5p	*MB*	ENSG00000198125	Coding RNA
Network10	hsa-miR-383-5p	*MEGF11*	ENSG00000157890	Coding RNA
Network10	hsa-miR-383-5p	*MLPH*	ENSG00000115648	Coding RNA
Network10	hsa-miR-383-5p	*MMP24*	ENSG00000125966	Coding RNA
Network10	hsa-miR-383-5p	*MYH7*	ENSG00000092054	Coding RNA
Network10	hsa-miR-383-5p	*MYO18B*	ENSG00000133454	Coding RNA
Network10	hsa-miR-383-5p	*MYT1*	ENSG00000196132	Coding RNA
Network10	hsa-miR-383-5p	*NCR3LG1*	ENSG00000188211	Coding RNA
Network10	hsa-miR-383-5p	*NGF*	ENSG00000134259	Coding RNA

Network10	hsa-miR-383-5p	*NOG*	ENSG00000183691	Coding RNA
Network10	hsa-miR-383-5p	*NOX4*	ENSG00000086991	Coding RNA
Network10	hsa-miR-383-5p	*P2RX3*	ENSG00000109991	Coding RNA
Network10	hsa-miR-383-5p	*PAX5*	ENSG00000196092	Coding RNA
Network10	hsa-miR-383-5p	*PRPH*	ENSG00000135406	Coding RNA
Network10	hsa-miR-383-5p	*PTGFR*	ENSG00000122420	Coding RNA
Network10	hsa-miR-383-5p	*PTGIS*	ENSG00000124212	Coding RNA
Network10	hsa-miR-383-5p	*RAB31*	ENSG00000168461	Coding RNA
Network10	hsa-miR-383-5p	*RASSF2*	ENSG00000101265	Coding RNA
Network10	hsa-miR-383-5p	*RORC*	ENSG00000143365	Coding RNA
Network10	hsa-miR-383-5p	*SCML2*	ENSG00000102098	Coding RNA
Network10	hsa-miR-383-5p	*SEZ6L2*	ENSG00000174938	Coding RNA
Network10	hsa-miR-383-5p	*SHISA3*	ENSG00000178343	Coding RNA
Network10	hsa-miR-383-5p	*SHISA9*	ENSG00000237515	Coding RNA
Network10	hsa-miR-383-5p	*SLC16A10*	ENSG00000112394	Coding RNA
Network10	hsa-miR-383-5p	*SLC35F1*	ENSG00000196376	Coding RNA
Network10	hsa-miR-383-5p	*SLC7A14*	ENSG00000013293	Coding RNA
Network10	hsa-miR-383-5p	*SLC8A3*	ENSG00000100678	Coding RNA
Network10	hsa-miR-383-5p	*SLIT1*	ENSG00000187122	Coding RNA
Network10	hsa-miR-383-5p	*SPRY4*	ENSG00000187678	Coding RNA
Network10	hsa-miR-383-5p	*ST6GAL2*	ENSG00000144057	Coding RNA
Network10	hsa-miR-383-5p	*STARD4*	ENSG00000164211	Coding RNA
Network10	hsa-miR-383-5p	*SUSD5*	ENSG00000173705	Coding RNA
Network10	hsa-miR-383-5p	*TFAP2B*	ENSG00000008196	Coding RNA
Network10	hsa-miR-383-5p	*TMC3*	ENSG00000188869	Coding RNA
Network10	hsa-miR-383-5p	*TMEM26*	ENSG00000196932	Coding RNA
Network10	hsa-miR-383-5p	*TMEM97*	ENSG00000109084	Coding RNA
Network10	hsa-miR-383-5p	*TNFRSF10B*	ENSG00000120889	Coding RNA
Network10	hsa-miR-383-5p	*TRIM67*	ENSG00000119283	Coding RNA
Network10	hsa-miR-383-5p	*UNC79*	ENSG00000133958	Coding RNA
Network10	hsa-miR-383-5p	*VWC2L*	ENSG00000174453	Coding RNA
Network10	hsa-miR-383-5p	*XYLT1*	ENSG00000103489	Coding RNA
Network10	hsa-miR-383-5p	*ZBTB8B*	ENSG00000273274	Coding RNA
Network10	hsa-miR-383-5p	*TBX5-AS1*	ENSG00000255399	lncRNA
Network11	hsa-miR-2114-3p	*ANO5*	ENSG00000171714	Coding RNA
Network11	hsa-miR-2114-3p	*CD5L*	ENSG00000073754	Coding RNA
Network11	hsa-miR-2114-3p	*CDHR1*	ENSG00000148600	Coding RNA
Network11	hsa-miR-2114-3p	*CELSR3*	ENSG00000008300	Coding RNA
Network11	hsa-miR-2114-3p	*CRISPLD2*	ENSG00000103196	Coding RNA
Network11	hsa-miR-2114-3p	*DUSP4*	ENSG00000120875	Coding RNA
Network11	hsa-miR-2114-3p	*ETV5*	ENSG00000244405	Coding RNA
Network11	hsa-miR-2114-3p	*G6PC2*	ENSG00000152254	Coding RNA
Network11	hsa-miR-2114-3p	*GFRA1*	ENSG00000151892	Coding RNA
Network11	hsa-miR-2114-3p	*KCNG4*	ENSG00000168418	Coding RNA
Network11	hsa-miR-2114-3p	*KCNK10*	ENSG00000100433	Coding RNA
Network11	hsa-miR-2114-3p	*KCNK3*	ENSG00000171303	Coding RNA
Network11	hsa-miR-2114-3p	*LPCAT1*	ENSG00000153395	Coding RNA
Network11	hsa-miR-2114-3p	*MEGF11*	ENSG00000157890	Coding RNA
Network11	hsa-miR-2114-3p	*MICAL1*	ENSG00000135596	Coding RNA
Network11	hsa-miR-2114-3p	*MLPH*	ENSG00000115648	Coding RNA
Network11	hsa-miR-2114-3p	*MMP24*	ENSG00000125966	Coding RNA
Network11	hsa-miR-2114-3p	*MYO3B*	ENSG00000071909	Coding RNA
Network11	hsa-miR-2114-3p	*NCR3LG1*	ENSG00000188211	Coding RNA
Network11	hsa-miR-2114-3p	*PAX5*	ENSG00000196092	Coding RNA
Network11	hsa-miR-2114-3p	*RHBDL3*	ENSG00000141314	Coding RNA
Network11	hsa-miR-2114-3p	*SALL4*	ENSG00000101115	Coding RNA

Network11	hsa-miR-2114-3p	*SCML2*	ENSG00000102098	Coding RNA
Network11	hsa-miR-2114-3p	*SLC18A1*	ENSG00000036565	Coding RNA
Network11	hsa-miR-2114-3p	*SPRY4*	ENSG00000187678	Coding RNA
Network11	hsa-miR-2114-3p	*TMC3*	ENSG00000188869	Coding RNA
Network11	hsa-miR-2114-3p	*TNFRSF10B*	ENSG00000120889	Coding RNA
Network11	hsa-miR-2114-3p	*TRIM67*	ENSG00000119283	Coding RNA
Network11	hsa-miR-2114-3p	*XKR7*	ENSG00000260903	Coding RNA
Network11	hsa-miR-2114-3p	*XYLT1*	ENSG00000103489	Coding RNA
Network11	hsa-miR-2114-3p	*LINC00654*	ENSG00000205181	lncRNA
Network11	hsa-miR-2114-3p	*LINC01132*	ENSG00000227630	lncRNA
Network12	hsa-miR-204-5p	*ABCC8*	ENSG00000006071	Coding RNA
Network12	hsa-miR-204-5p	*ACAN*	ENSG00000157766	Coding RNA
Network12	hsa-miR-204-5p	*ACTC1*	ENSG00000159251	Coding RNA
Network12	hsa-miR-204-5p	*AEN*	ENSG00000181026	Coding RNA
Network12	hsa-miR-204-5p	*AHSG*	ENSG00000145192	Coding RNA
Network12	hsa-miR-204-5p	*ALK*	ENSG00000171094	Coding RNA
Network12	hsa-miR-204-5p	*AMZ1*	ENSG00000174945	Coding RNA
Network12	hsa-miR-204-5p	*ANK1*	ENSG00000029534	Coding RNA
Network12	hsa-miR-204-5p	*ANO5*	ENSG00000171714	Coding RNA
Network12	hsa-miR-204-5p	*ASIC4*	ENSG00000072182	Coding RNA
Network12	hsa-miR-204-5p	*ATOH8*	ENSG00000168874	Coding RNA
Network12	hsa-miR-204-5p	*ATRX*	ENSG00000085224	Coding RNA
Network12	hsa-miR-204-5p	*BLK*	ENSG00000136573	Coding RNA
Network12	hsa-miR-204-5p	*C1QTNF1*	ENSG00000173918	Coding RNA
Network12	hsa-miR-204-5p	*C20orf204*	ENSG00000196421	Coding RNA
Network12	hsa-miR-204-5p	*CA10*	ENSG00000154975	Coding RNA
Network12	hsa-miR-204-5p	*CACNG2*	ENSG00000166862	Coding RNA
Network12	hsa-miR-204-5p	*CACNG4*	ENSG00000075461	Coding RNA
Network12	hsa-miR-204-5p	*CAPN14*	ENSG00000214711	Coding RNA
Network12	hsa-miR-204-5p	*CCSER1*	ENSG00000184305	Coding RNA
Network12	hsa-miR-204-5p	*CDKN1A*	ENSG00000124762	Coding RNA
Network12	hsa-miR-204-5p	*CELSR3*	ENSG00000008300	Coding RNA
Network12	hsa-miR-204-5p	*CHGB*	ENSG00000089199	Coding RNA
Network12	hsa-miR-204-5p	*CLEC4F*	ENSG00000152672	Coding RNA
Network12	hsa-miR-204-5p	*CLVS1*	ENSG00000177182	Coding RNA
Network12	hsa-miR-204-5p	*CMBL*	ENSG00000164237	Coding RNA
Network12	hsa-miR-204-5p	*CMKLR1*	ENSG00000174600	Coding RNA
Network12	hsa-miR-204-5p	*COL17A1*	ENSG00000065618	Coding RNA
Network12	hsa-miR-204-5p	*COL4A3*	ENSG00000169031	Coding RNA
Network12	hsa-miR-204-5p	*COL4A4*	ENSG00000081052	Coding RNA
Network12	hsa-miR-204-5p	*COL9A1*	ENSG00000112280	Coding RNA
Network12	hsa-miR-204-5p	*CPA5*	ENSG00000158525	Coding RNA
Network12	hsa-miR-204-5p	*CREB3L4*	ENSG00000143578	Coding RNA
Network12	hsa-miR-204-5p	*CSPG5*	ENSG00000114646	Coding RNA
Network12	hsa-miR-204-5p	*DGKG*	ENSG00000058866	Coding RNA
Network12	hsa-miR-204-5p	*DHRS2*	ENSG00000100867	Coding RNA
Network12	hsa-miR-204-5p	*DLX3*	ENSG00000064195	Coding RNA
Network12	hsa-miR-204-5p	*DNAH14*	ENSG00000185842	Coding RNA
Network12	hsa-miR-204-5p	*DNASE1L3*	ENSG00000163687	Coding RNA
Network12	hsa-miR-204-5p	*DRAM1*	ENSG00000136048	Coding RNA
Network12	hsa-miR-204-5p	*DRG2*	ENSG00000108591	Coding RNA
Network12	hsa-miR-204-5p	*DUSP4*	ENSG00000120875	Coding RNA
Network12	hsa-miR-204-5p	*DUSP6*	ENSG00000139318	Coding RNA
Network12	hsa-miR-204-5p	*EPN2*	ENSG00000072134	Coding RNA
Network12	hsa-miR-204-5p	*ESRRG*	ENSG00000196482	Coding RNA
Network12	hsa-miR-204-5p	*ETV4*	ENSG00000175832	Coding RNA

Network12	hsa-miR-204-5p	*ETV5*	ENSG00000244405	Coding RNA
Network12	hsa-miR-204-5p	*FAM155B*	ENSG00000130054	Coding RNA
Network12	hsa-miR-204-5p	*FDXR*	ENSG00000161513	Coding RNA
Network12	hsa-miR-204-5p	*FGFBP3*	ENSG00000174721	Coding RNA
Network12	hsa-miR-204-5p	*FOXF2*	ENSG00000137273	Coding RNA
Network12	hsa-miR-204-5p	*FRMPD1*	ENSG00000070601	Coding RNA
Network12	hsa-miR-204-5p	*G6PC2*	ENSG00000152254	Coding RNA
Network12	hsa-miR-204-5p	*GALR1*	ENSG00000166573	Coding RNA
Network12	hsa-miR-204-5p	*GCNT2*	ENSG00000111846	Coding RNA
Network12	hsa-miR-204-5p	*GFRA1*	ENSG00000151892	Coding RNA
Network12	hsa-miR-204-5p	*GLRA1*	ENSG00000145888	Coding RNA
Network12	hsa-miR-204-5p	*GRID2IP*	ENSG00000215045	Coding RNA
Network12	hsa-miR-204-5p	*INVS*	ENSG00000119509	Coding RNA
Network12	hsa-miR-204-5p	*KCNH7*	ENSG00000184611	Coding RNA
Network12	hsa-miR-204-5p	*KCNIP2*	ENSG00000120049	Coding RNA
Network12	hsa-miR-204-5p	*KCNK3*	ENSG00000171303	Coding RNA
Network12	hsa-miR-204-5p	*KIF26B*	ENSG00000162849	Coding RNA
Network12	hsa-miR-204-5p	*KLRC1*	ENSG00000134545	Coding RNA
Network12	hsa-miR-204-5p	*L1CAM*	ENSG00000198910	Coding RNA
Network12	hsa-miR-204-5p	*LEMD1*	ENSG00000186007	Coding RNA
Network12	hsa-miR-204-5p	*LHFPL3*	ENSG00000187416	Coding RNA
Network12	hsa-miR-204-5p	*LHX5*	ENSG00000089116	Coding RNA
Network12	hsa-miR-204-5p	*LINGO4*	ENSG00000213171	Coding RNA
Network12	hsa-miR-204-5p	*LPCAT1*	ENSG00000153395	Coding RNA
Network12	hsa-miR-204-5p	*LPO*	ENSG00000167419	Coding RNA
Network12	hsa-miR-204-5p	*LRP4*	ENSG00000134569	Coding RNA
Network12	hsa-miR-204-5p	*LRRC74A*	ENSG00000100565	Coding RNA
Network12	hsa-miR-204-5p	*MEGF11*	ENSG00000157890	Coding RNA
Network12	hsa-miR-204-5p	*MMP24*	ENSG00000125966	Coding RNA
Network12	hsa-miR-204-5p	*MYH7*	ENSG00000092054	Coding RNA
Network12	hsa-miR-204-5p	*MYO18B*	ENSG00000133454	Coding RNA
Network12	hsa-miR-204-5p	*MYO3B*	ENSG00000071909	Coding RNA
Network12	hsa-miR-204-5p	*MYT1*	ENSG00000196132	Coding RNA
Network12	hsa-miR-204-5p	*NCAN*	ENSG00000130287	Coding RNA
Network12	hsa-miR-204-5p	*NCR3LG1*	ENSG00000188211	Coding RNA
Network12	hsa-miR-204-5p	*NEUROD4*	ENSG00000123307	Coding RNA
Network12	hsa-miR-204-5p	*NGFR*	ENSG00000064300	Coding RNA
Network12	hsa-miR-204-5p	*NMU*	ENSG00000109255	Coding RNA
Network12	hsa-miR-204-5p	*NOG*	ENSG00000183691	Coding RNA
Network12	hsa-miR-204-5p	*NOX4*	ENSG00000086991	Coding RNA
Network12	hsa-miR-204-5p	*P2RX3*	ENSG00000109991	Coding RNA
Network12	hsa-miR-204-5p	*PAX5*	ENSG00000196092	Coding RNA
Network12	hsa-miR-204-5p	*PRCD*	ENSG00000214140	Coding RNA
Network12	hsa-miR-204-5p	*PTGIS*	ENSG00000124212	Coding RNA
Network12	hsa-miR-204-5p	*RAB31*	ENSG00000168461	Coding RNA
Network12	hsa-miR-204-5p	*RASSF2*	ENSG00000101265	Coding RNA
Network12	hsa-miR-204-5p	*RHBDL3*	ENSG00000141314	Coding RNA
Network12	hsa-miR-204-5p	*RPL22L1*	ENSG00000163584	Coding RNA
Network12	hsa-miR-204-5p	*RUFY4*	ENSG00000188282	Coding RNA
Network12	hsa-miR-204-5p	*SALL4*	ENSG00000101115	Coding RNA
Network12	hsa-miR-204-5p	*SAPCD2*	ENSG00000186193	Coding RNA
Network12	hsa-miR-204-5p	*SCARA5*	ENSG00000168079	Coding RNA
Network12	hsa-miR-204-5p	*SCEL*	ENSG00000136155	Coding RNA
Network12	hsa-miR-204-5p	*SCML2*	ENSG00000102098	Coding RNA
Network12	hsa-miR-204-5p	*SCRT2*	ENSG00000215397	Coding RNA
Network12	hsa-miR-204-5p	*SEZ6*	ENSG00000063015	Coding RNA

Network12	hsa-miR-204-5p	*SEZ6L*	ENSG00000100095	Coding RNA
Network12	hsa-miR-204-5p	*SEZ6L2*	ENSG00000174938	Coding RNA
Network12	hsa-miR-204-5p	*SHC3*	ENSG00000148082	Coding RNA
Network12	hsa-miR-204-5p	*SHISA9*	ENSG00000237515	Coding RNA
Network12	hsa-miR-204-5p	*SKOR2*	ENSG00000215474	Coding RNA
Network12	hsa-miR-204-5p	*SLAIN1*	ENSG00000139737	Coding RNA
Network12	hsa-miR-204-5p	*SLC16A10*	ENSG00000112394	Coding RNA
Network12	hsa-miR-204-5p	*SLC17A8*	ENSG00000179520	Coding RNA
Network12	hsa-miR-204-5p	*SLC18A1*	ENSG00000036565	Coding RNA
Network12	hsa-miR-204-5p	*SLC35F1*	ENSG00000196376	Coding RNA
Network12	hsa-miR-204-5p	*SLC7A14*	ENSG00000013293	Coding RNA
Network12	hsa-miR-204-5p	*SLC8A3*	ENSG00000100678	Coding RNA
Network12	hsa-miR-204-5p	*SLIT1*	ENSG00000187122	Coding RNA
Network12	hsa-miR-204-5p	*SPRY4*	ENSG00000187678	Coding RNA
Network12	hsa-miR-204-5p	*SRRM3*	ENSG00000177679	Coding RNA
Network12	hsa-miR-204-5p	*ST6GAL2*	ENSG00000144057	Coding RNA
Network12	hsa-miR-204-5p	*STARD4*	ENSG00000164211	Coding RNA
Network12	hsa-miR-204-5p	*SUSD5*	ENSG00000173705	Coding RNA
Network12	hsa-miR-204-5p	*SYCE2*	ENSG00000161860	Coding RNA
Network12	hsa-miR-204-5p	*TFAP2B*	ENSG00000008196	Coding RNA
Network12	hsa-miR-204-5p	*TLX1*	ENSG00000107807	Coding RNA
Network12	hsa-miR-204-5p	*TMEM26*	ENSG00000196932	Coding RNA
Network12	hsa-miR-204-5p	*TMEM97*	ENSG00000109084	Coding RNA
Network12	hsa-miR-204-5p	*TNFRSF10B*	ENSG00000120889	Coding RNA
Network12	hsa-miR-204-5p	*TNR*	ENSG00000116147	Coding RNA
Network12	hsa-miR-204-5p	*TOGARAM2*	ENSG00000189350	Coding RNA
Network12	hsa-miR-204-5p	*TRIM67*	ENSG00000119283	Coding RNA
Network12	hsa-miR-204-5p	*TRMT9B*	ENSG00000250305	Coding RNA
Network12	hsa-miR-204-5p	*VASH2*	ENSG00000143494	Coding RNA
Network12	hsa-miR-204-5p	*XKR7*	ENSG00000260903	Coding RNA
Network12	hsa-miR-204-5p	*XYLT1*	ENSG00000103489	Coding RNA
Network12	hsa-miR-204-5p	*ZBTB8B*	ENSG00000273274	Coding RNA
Network12	hsa-miR-204-5p	*ZDHHC22*	ENSG00000177108	Coding RNA
Network12	hsa-miR-204-5p	*ZFR2*	ENSG00000105278	Coding RNA
Network12	hsa-miR-204-5p	*ZNF257*	ENSG00000197134	Coding RNA
Network12	hsa-miR-204-5p	*ZNF676*	ENSG00000196109	Coding RNA
Network12	hsa-miR-204-5p	*EPN2-AS1*	ENSG00000235397	lncRNA
Network12	hsa-miR-204-5p	*MROCKI*	ENSG00000227502	lncRNA
Network12	hsa-miR-204-5p	*SMCR5*	ENSG00000226746	lncRNA
Network12	hsa-miR-204-5p	*TBX5-AS1*	ENSG00000255399	lncRNA
Network12	hsa-miR-204-5p	*TM4SF19-AS1*	ENSG00000235897	lncRNA
Network12	hsa-miR-204-5p	*GUCY1B2*	ENSG00000123201	Pseudogene
Network12	hsa-miR-204-5p	*NXF4*	ENSG00000196970	Pseudogene
Network13	hsa-miR-7156-5p	*AEN*	ENSG00000181026	Coding RNA
Network13	hsa-miR-7156-5p	*ARL4A*	ENSG00000122644	Coding RNA
Network13	hsa-miR-7156-5p	*CA10*	ENSG00000154975	Coding RNA
Network13	hsa-miR-7156-5p	*CACNG2*	ENSG00000166862	Coding RNA
Network13	hsa-miR-7156-5p	*CAPN14*	ENSG00000214711	Coding RNA
Network13	hsa-miR-7156-5p	*CD5L*	ENSG00000073754	Coding RNA
Network13	hsa-miR-7156-5p	*CELSR3*	ENSG00000008300	Coding RNA
Network13	hsa-miR-7156-5p	*CLVS1*	ENSG00000177182	Coding RNA
Network13	hsa-miR-7156-5p	*CMKLR1*	ENSG00000174600	Coding RNA
Network13	hsa-miR-7156-5p	*DHRS2*	ENSG00000100867	Coding RNA
Network13	hsa-miR-7156-5p	*INSM2*	ENSG00000168348	Coding RNA
Network13	hsa-miR-7156-5p	*KCNIP2*	ENSG00000120049	Coding RNA
Network13	hsa-miR-7156-5p	*LPCAT1*	ENSG00000153395	Coding RNA

Network13	hsa-miR-7156-5p	*MLPH*	ENSG00000115648	Coding RNA
Network13	hsa-miR-7156-5p	*MMD2*	ENSG00000136297	Coding RNA
Network13	hsa-miR-7156-5p	*NOG*	ENSG00000183691	Coding RNA
Network13	hsa-miR-7156-5p	*PTGFR*	ENSG00000122420	Coding RNA
Network13	hsa-miR-7156-5p	*RHBDL3*	ENSG00000141314	Coding RNA
Network13	hsa-miR-7156-5p	*RIT2*	ENSG00000152214	Coding RNA
Network13	hsa-miR-7156-5p	*SCML2*	ENSG00000102098	Coding RNA
Network13	hsa-miR-7156-5p	*SEZ6*	ENSG00000063015	Coding RNA
Network13	hsa-miR-7156-5p	*SHISA3*	ENSG00000178343	Coding RNA
Network13	hsa-miR-7156-5p	*SHISA9*	ENSG00000237515	Coding RNA
Network13	hsa-miR-7156-5p	*SPRY4*	ENSG00000187678	Coding RNA
Network13	hsa-miR-7156-5p	*ST6GAL2*	ENSG00000144057	Coding RNA
Network13	hsa-miR-7156-5p	*TBX5*	ENSG00000089225	Coding RNA
Network13	hsa-miR-7156-5p	*TFAP2B*	ENSG00000008196	Coding RNA
Network13	hsa-miR-7156-5p	*TMEM26*	ENSG00000196932	Coding RNA
Network13	hsa-miR-7156-5p	*ZFR2*	ENSG00000105278	Coding RNA
Network13	hsa-miR-7156-5p	*ZNF208*	ENSG00000160321	Coding RNA
Network13	hsa-miR-7156-5p	*ZNF257*	ENSG00000197134	Coding RNA
Network13	hsa-miR-7156-5p	*INHBA-AS1*	ENSG00000224116	lncRNA
Network13	hsa-miR-7156-5p	*LINC00654*	ENSG00000205181	lncRNA
Network13	hsa-miR-7156-5p	*LINC01132*	ENSG00000227630	lncRNA
Network13	hsa-miR-7156-5p	*LINC01411*	ENSG00000249306	lncRNA
Network13	hsa-miR-7156-5p	*LINC01551*	ENSG00000186960	lncRNA
Network13	hsa-miR-7156-5p	*MROCKI*	ENSG00000227502	lncRNA
Network14	hsa-miR-92b-3p	*ACAN*	ENSG00000157766	Coding RNA
Network14	hsa-miR-92b-3p	*ACAT2*	ENSG00000120437	Coding RNA
Network14	hsa-miR-92b-3p	*ACTC1*	ENSG00000159251	Coding RNA
Network14	hsa-miR-92b-3p	*AEN*	ENSG00000181026	Coding RNA
Network14	hsa-miR-92b-3p	*ARL4A*	ENSG00000122644	Coding RNA
Network14	hsa-miR-92b-3p	*ATRX*	ENSG00000085224	Coding RNA
Network14	hsa-miR-92b-3p	*CCSER1*	ENSG00000184305	Coding RNA
Network14	hsa-miR-92b-3p	*CDKN1A*	ENSG00000124762	Coding RNA
Network14	hsa-miR-92b-3p	*CELSR3*	ENSG00000008300	Coding RNA
Network14	hsa-miR-92b-3p	*CLVS1*	ENSG00000177182	Coding RNA
Network14	hsa-miR-92b-3p	*CMBL*	ENSG00000164237	Coding RNA
Network14	hsa-miR-92b-3p	*CMKLR1*	ENSG00000174600	Coding RNA
Network14	hsa-miR-92b-3p	*COL17A1*	ENSG00000065618	Coding RNA
Network14	hsa-miR-92b-3p	*COL4A3*	ENSG00000169031	Coding RNA
Network14	hsa-miR-92b-3p	*COL4A4*	ENSG00000081052	Coding RNA
Network14	hsa-miR-92b-3p	*CRISPLD2*	ENSG00000103196	Coding RNA
Network14	hsa-miR-92b-3p	*CXCL13*	ENSG00000156234	Coding RNA
Network14	hsa-miR-92b-3p	*DAND5*	ENSG00000179284	Coding RNA
Network14	hsa-miR-92b-3p	*DLX3*	ENSG00000064195	Coding RNA
Network14	hsa-miR-92b-3p	*DRAM1*	ENSG00000136048	Coding RNA
Network14	hsa-miR-92b-3p	*DRG2*	ENSG00000108591	Coding RNA
Network14	hsa-miR-92b-3p	*DUSP4*	ENSG00000120875	Coding RNA
Network14	hsa-miR-92b-3p	*DUSP6*	ENSG00000139318	Coding RNA
Network14	hsa-miR-92b-3p	*EPN2*	ENSG00000072134	Coding RNA
Network14	hsa-miR-92b-3p	*ESRRG*	ENSG00000196482	Coding RNA
Network14	hsa-miR-92b-3p	*ETV4*	ENSG00000175832	Coding RNA
Network14	hsa-miR-92b-3p	*ETV5*	ENSG00000244405	Coding RNA
Network14	hsa-miR-92b-3p	*FAM155B*	ENSG00000130054	Coding RNA
Network14	hsa-miR-92b-3p	*FGFBP3*	ENSG00000174721	Coding RNA
Network14	hsa-miR-92b-3p	*GLRA1*	ENSG00000145888	Coding RNA
Network14	hsa-miR-92b-3p	*IL11RA*	ENSG00000137070	Coding RNA
Network14	hsa-miR-92b-3p	*INSM2*	ENSG00000168348	Coding RNA

Network14	hsa-miR-92b-3p	*INSYN2A*	ENSG00000188916	Coding RNA
Network14	hsa-miR-92b-3p	*KCNIP2*	ENSG00000120049	Coding RNA
Network14	hsa-miR-92b-3p	*KCNJ11*	ENSG00000187486	Coding RNA
Network14	hsa-miR-92b-3p	*KCNK10*	ENSG00000100433	Coding RNA
Network14	hsa-miR-92b-3p	*KCNK3*	ENSG00000171303	Coding RNA
Network14	hsa-miR-92b-3p	*LHFPL3*	ENSG00000187416	Coding RNA
Network14	hsa-miR-92b-3p	*LINGO4*	ENSG00000213171	Coding RNA
Network14	hsa-miR-92b-3p	*LPCAT1*	ENSG00000153395	Coding RNA
Network14	hsa-miR-92b-3p	*LRRC74A*	ENSG00000100565	Coding RNA
Network14	hsa-miR-92b-3p	*MB*	ENSG00000198125	Coding RNA
Network14	hsa-miR-92b-3p	*MYO3B*	ENSG00000071909	Coding RNA
Network14	hsa-miR-92b-3p	*MYT1*	ENSG00000196132	Coding RNA
Network14	hsa-miR-92b-3p	*NAT16*	ENSG00000167011	Coding RNA
Network14	hsa-miR-92b-3p	*NCAN*	ENSG00000130287	Coding RNA
Network14	hsa-miR-92b-3p	*NOX4*	ENSG00000086991	Coding RNA
Network14	hsa-miR-92b-3p	*PAX5*	ENSG00000196092	Coding RNA
Network14	hsa-miR-92b-3p	*PTGFR*	ENSG00000122420	Coding RNA
Network14	hsa-miR-92b-3p	*RAB31*	ENSG00000168461	Coding RNA
Network14	hsa-miR-92b-3p	*RASSF2*	ENSG00000101265	Coding RNA
Network14	hsa-miR-92b-3p	*RLBP1*	ENSG00000140522	Coding RNA
Network14	hsa-miR-92b-3p	*RORC*	ENSG00000143365	Coding RNA
Network14	hsa-miR-92b-3p	*RPL22L1*	ENSG00000163584	Coding RNA
Network14	hsa-miR-92b-3p	*SALL4*	ENSG00000101115	Coding RNA
Network14	hsa-miR-92b-3p	*SCML2*	ENSG00000102098	Coding RNA
Network14	hsa-miR-92b-3p	*SCRT2*	ENSG00000215397	Coding RNA
Network14	hsa-miR-92b-3p	*SLAIN1*	ENSG00000139737	Coding RNA
Network14	hsa-miR-92b-3p	*SLC16A10*	ENSG00000112394	Coding RNA
Network14	hsa-miR-92b-3p	*SLC18A1*	ENSG00000036565	Coding RNA
Network14	hsa-miR-92b-3p	*SLC39A2*	ENSG00000165794	Coding RNA
Network14	hsa-miR-92b-3p	*ST6GAL2*	ENSG00000144057	Coding RNA
Network14	hsa-miR-92b-3p	*STARD4*	ENSG00000164211	Coding RNA
Network14	hsa-miR-92b-3p	*SUSD5*	ENSG00000173705	Coding RNA
Network14	hsa-miR-92b-3p	*TFAP2B*	ENSG00000008196	Coding RNA
Network14	hsa-miR-92b-3p	*TLX1*	ENSG00000107807	Coding RNA
Network14	hsa-miR-92b-3p	*TMEM26*	ENSG00000196932	Coding RNA
Network14	hsa-miR-92b-3p	*TMEM97*	ENSG00000109084	Coding RNA
Network14	hsa-miR-92b-3p	*TNFRSF10B*	ENSG00000120889	Coding RNA
Network14	hsa-miR-92b-3p	*UNC79*	ENSG00000133958	Coding RNA
Network14	hsa-miR-92b-3p	*VASH2*	ENSG00000143494	Coding RNA
Network14	hsa-miR-92b-3p	*XKR7*	ENSG00000260903	Coding RNA
Network14	hsa-miR-92b-3p	*ZBTB8B*	ENSG00000273274	Coding RNA
Network14	hsa-miR-92b-3p	*ZFR2*	ENSG00000105278	Coding RNA
Network14	hsa-miR-92b-3p	*ZNF208*	ENSG00000160321	Coding RNA
Network14	hsa-miR-92b-3p	*ZNF99*	ENSG00000213973	Coding RNA
Network14	hsa-miR-92b-3p	*CHL1-AS1*	ENSG00000234661	lncRNA
Network14	hsa-miR-92b-3p	*LINC01551*	ENSG00000186960	lncRNA
Network14	hsa-miR-92b-3p	*TBX5-AS1*	ENSG00000255399	lncRNA
Network14	hsa-miR-92b-3p	*LPAL2*	ENSG00000213071	Pseudogene
Network15	hsa-miR-3074-5p	*ACAN*	ENSG00000157766	Coding RNA
Network15	hsa-miR-3074-5p	*ANK1*	ENSG00000029534	Coding RNA
Network15	hsa-miR-3074-5p	*ATCAY*	ENSG00000167654	Coding RNA
Network15	hsa-miR-3074-5p	*ATOH8*	ENSG00000168874	Coding RNA
Network15	hsa-miR-3074-5p	*CA10*	ENSG00000154975	Coding RNA
Network15	hsa-miR-3074-5p	*CCSER1*	ENSG00000184305	Coding RNA
Network15	hsa-miR-3074-5p	*CMKLR1*	ENSG00000174600	Coding RNA
Network15	hsa-miR-3074-5p	*DGKG*	ENSG00000058866	Coding RNA

Network15	hsa-miR-3074-5p	*DRAM1*	ENSG00000136048	Coding RNA
Network15	hsa-miR-3074-5p	*FAM155B*	ENSG00000130054	Coding RNA
Network15	hsa-miR-3074-5p	*FGFBP3*	ENSG00000174721	Coding RNA
Network15	hsa-miR-3074-5p	*GFRA1*	ENSG00000151892	Coding RNA
Network15	hsa-miR-3074-5p	*GLRA1*	ENSG00000145888	Coding RNA
Network15	hsa-miR-3074-5p	*HCAR1*	ENSG00000196917	Coding RNA
Network15	hsa-miR-3074-5p	*KCNK10*	ENSG00000100433	Coding RNA
Network15	hsa-miR-3074-5p	*KCNK3*	ENSG00000171303	Coding RNA
Network15	hsa-miR-3074-5p	*MMD2*	ENSG00000136297	Coding RNA
Network15	hsa-miR-3074-5p	*MYO18B*	ENSG00000133454	Coding RNA
Network15	hsa-miR-3074-5p	*P2RX3*	ENSG00000109991	Coding RNA
Network15	hsa-miR-3074-5p	*PRCD*	ENSG00000214140	Coding RNA
Network15	hsa-miR-3074-5p	*PTGIS*	ENSG00000124212	Coding RNA
Network15	hsa-miR-3074-5p	*RAB31*	ENSG00000168461	Coding RNA
Network15	hsa-miR-3074-5p	*RHBDL3*	ENSG00000141314	Coding RNA
Network15	hsa-miR-3074-5p	*SALL4*	ENSG00000101115	Coding RNA
Network15	hsa-miR-3074-5p	*SEZ6L*	ENSG00000100095	Coding RNA
Network15	hsa-miR-3074-5p	*SLC16A10*	ENSG00000112394	Coding RNA
Network15	hsa-miR-3074-5p	*SLC39A2*	ENSG00000165794	Coding RNA
Network15	hsa-miR-3074-5p	*SPRY4*	ENSG00000187678	Coding RNA
Network15	hsa-miR-3074-5p	*TNFRSF10B*	ENSG00000120889	Coding RNA
Network15	hsa-miR-3074-5p	*TRIM67*	ENSG00000119283	Coding RNA
Network15	hsa-miR-3074-5p	*XYLT1*	ENSG00000103489	Coding RNA
Network15	hsa-miR-3074-5p	*CD81-AS1*	ENSG00000238184	lncRNA
Network15	hsa-miR-3074-5p	*CHL1-AS1*	ENSG00000234661	lncRNA
Network15	hsa-miR-3074-5p	*FBXL19-AS1*	ENSG00000260852	lncRNA
Network15	hsa-miR-3074-5p	*FZD10-AS1*	ENSG00000250208	lncRNA
Network15	hsa-miR-3074-5p	*INHBA-AS1*	ENSG00000224116	lncRNA
Network15	hsa-miR-3074-5p	*LINC00574*	ENSG00000231690	lncRNA
Network15	hsa-miR-3074-5p	*LINC01132*	ENSG00000227630	lncRNA
Network15	hsa-miR-3074-5p	*LINC01497*	ENSG00000237560	lncRNA
Network15	hsa-miR-3074-5p	*LINC01623*	ENSG00000225595	lncRNA
Network16	hsa-miR-1298-3p	*ALK*	ENSG00000171094	Coding RNA
Network16	hsa-miR-1298-3p	*ATRX*	ENSG00000085224	Coding RNA
Network16	hsa-miR-1298-3p	*CAPN14*	ENSG00000214711	Coding RNA
Network16	hsa-miR-1298-3p	*CCSER1*	ENSG00000184305	Coding RNA
Network16	hsa-miR-1298-3p	*COL17A1*	ENSG00000065618	Coding RNA
Network16	hsa-miR-1298-3p	*DRG2*	ENSG00000108591	Coding RNA
Network16	hsa-miR-1298-3p	*FAM155B*	ENSG00000130054	Coding RNA
Network16	hsa-miR-1298-3p	*G6PC2*	ENSG00000152254	Coding RNA
Network16	hsa-miR-1298-3p	*GFRA1*	ENSG00000151892	Coding RNA
Network16	hsa-miR-1298-3p	*INVS*	ENSG00000119509	Coding RNA
Network16	hsa-miR-1298-3p	*KCNIP2*	ENSG00000120049	Coding RNA
Network16	hsa-miR-1298-3p	*KCNK10*	ENSG00000100433	Coding RNA
Network16	hsa-miR-1298-3p	*KCNK3*	ENSG00000171303	Coding RNA
Network16	hsa-miR-1298-3p	*KIF26B*	ENSG00000162849	Coding RNA
Network16	hsa-miR-1298-3p	*P2RX3*	ENSG00000109991	Coding RNA
Network16	hsa-miR-1298-3p	*PAX5*	ENSG00000196092	Coding RNA
Network16	hsa-miR-1298-3p	*PTGFR*	ENSG00000122420	Coding RNA
Network16	hsa-miR-1298-3p	*RORC*	ENSG00000143365	Coding RNA
Network16	hsa-miR-1298-3p	*SCML2*	ENSG00000102098	Coding RNA
Network16	hsa-miR-1298-3p	*SHC3*	ENSG00000148082	Coding RNA
Network16	hsa-miR-1298-3p	*SHISA9*	ENSG00000237515	Coding RNA
Network16	hsa-miR-1298-3p	*SLIT1*	ENSG00000187122	Coding RNA
Network16	hsa-miR-1298-3p	*SRRM3*	ENSG00000177679	Coding RNA
Network16	hsa-miR-1298-3p	*STARD4*	ENSG00000164211	Coding RNA

Network16	hsa-miR-1298-3p	*SUSD5*	ENSG00000173705	Coding RNA
Network16	hsa-miR-1298-3p	*SYCE1*	ENSG00000171772	Coding RNA
Network16	hsa-miR-1298-3p	*TMEM97*	ENSG00000109084	Coding RNA
Network16	hsa-miR-1298-3p	*TRIM67*	ENSG00000119283	Coding RNA
Network16	hsa-miR-1298-3p	*TRMT9B*	ENSG00000250305	Coding RNA
Network16	hsa-miR-1298-3p	*ZDHHC22*	ENSG00000177108	Coding RNA
Network16	hsa-miR-1298-3p	*CISTR*	ENSG00000260492	lncRNA
Network16	hsa-miR-1298-3p	*LINC01551*	ENSG00000186960	lncRNA

Table S3. Results of GO and pathway analysis.

GO Category	Functional Category	Count	FDR	Genes
GO BP	Regulation of biological quality	71	0.003	*CACNG4 CETP PNPLA3 KCNK10 PTGFR FAM155B NGF TRPC3 GLRA1 ACE TERT CACNG2 TRH KCNK3 CMKLR1 DAND5 SLC17A8 KCNH7 SHISA9 XKR7 HCN4 MYH7 TMEM97 KCNIP2 HCRTR1 PTGIS HIP1R PDX1 CXCL13 STARD4 C1QTNF1 FGFBP3 KCNJ11 INSC ABCC8 TFAP2B SLC18A1 SEZ6 ATRX NOX4 SLC8A3 RASSF2 NMU P2RX3 SLC16A10 CSPG5 TNR LRP4 CDHR1 LPCAT1 GJD2 GALR1 SCARA5 ESRRG MB GRID2IP ETV5 GCNT2 BLK RIT2 G6PC2 NGFR SLIT1 L1CAM DHRS2 DGKG CDKN1A MICAL1 FDXR LPO DGKK*
	Cell differentiation	67	0.027	*COL4A4 LHX5 TNNT1 MNX1 NGF BLK PDX1 DNASE1L3 NKX6-3 ALK ETV4 NOG LOR SKOR2 ETV5 CETP EDA2R ACE TFAP2B TBX5 KCNIP2 SCEL MMD2 ATOH8 SLIT1 L1CAM ABCC8 CELSR3 CHRDL2 DGKG SEZ6 NGFR ATRX NOX4 SLC8A3 RASSF2 CSPG5 TNR TRIM67 ARL4A CDKN1A BARHL1 MMP24 MYO18B LRP4 DUSP6 RIT2 ACAN ACTC1 TERT ATCAY VWC2L CMKLR1 INSC PAX5 MYT1 MB SCRT2 SCRT1 DHRS2 GCNT2 CDHR1 RORC CREB3L1 FOXF2 SHC3 GFRA1*
	Regulation of multicellular organismal process	57	0.009	*NMU NGF DUSP6 ALK DAND5 ACE TBX5 MYH7 TNNT1 MMD2 TRPC3 VASH2 CXCL13 ATOH8 COL4A3 C1QTNF1 NOG ABCC8 CELSR3 SEZ6 CACNG4 LHX5 SLC8A3 RASSF2 P2RX3 TNR TRIM67 HIP1R KREMEN2 LRP4 HCN4 AHSG GLRA1 RIT2 TERT VWC2L CMKLR1 SAPCD2 ESRRG SCRT2 SKOR2 SHISA9 ETV5 SCRT1 NGFR CREB3L1 EPN2 GCNT2 PTGIS GALR1 SLIT1 L1CAM KCNIP2 CACNG2 KCNK3 KCNIP1 KCNJ11*
	Nervous system development	47	0.003	*LHX5 BARHL1 NCAN MNX1 NGF GFRA1 ACAN ALK SKOR2 KCNIP2 MMD2 SLIT1 L1CAM ABCC8 TFAP2B CELSR3 DGKG SEZ6 NGFR ATRX SEZ6L SLC8A3 SALL4 CSPG5 TNR TRIM67 MMP24 LRP4 PDX1 SHC3 RIT2 TERT ATCAY ATOH8 KCNK3 VWC2L SEZ6L2 SLC17A8 NOG INSC PAX5 MYT1 SCRT2 ETV5 SCRT1 CDHR1 CA10*
	Cell-cell signaling	41	0.001	*CACNG4 NMU NGF GLRA1 CACNG2 TRH FGFBP3 SLC17A8 SHISA9 TBX5 INVS LRP4 PDX1 CXCL13 C1QTNF1 NOG ABCC8*

			CELSR3 SEZ6 LHX5 SLC8A3 P2RX3 CSPG5 TNR KREMEN2 SHC3 GJD2 TERT GRID2IP ETV5 TFAP2B SCEL BLK HCN4 G6PC2 GALR1 KCNJ11 KCNIP2 HCRTR1 RIT2 KCNK3
Regulation of system process	21	0.001	*NMU ACE MYH7 TNNT1 TRPC3 C1QTNF1 ABCC8 CACNG4 SLC8A3 P2RX3 TNR HIP1R HCN4 GLRA1 SHISA9 GALR1 KCNIP2 CACNG2 KCNK3 KCNIP1 KCNJ11*
Anterograde trans-synaptic signaling	20	0.010	*CACNG4 NMU NGF GLRA1 CACNG2 SLC17A8 SHISA9 SEZ6 SLC8A3 P2RX3 CSPG5 TNR SHC3 GJD2 GRID2IP ETV5 KCNIP2 HCRTR1 RIT2 KCNK3*
Chemical synaptic transmission	20	0.010	*CACNG4 NMU NGF GLRA1 CACNG2 SLC17A8 SHISA9 SEZ6 SLC8A3 P2RX3 CSPG5 TNR SHC3 GJD2 GRID2IP ETV5 KCNIP2 HCRTR1 RIT2 KCNK3*
Trans-synaptic signaling	20	0.011	*CACNG4 NMU NGF GLRA1 CACNG2 SLC17A8 SHISA9 SEZ6 SLC8A3 P2RX3 CSPG5 TNR SHC3 GJD2 GRID2IP ETV5 KCNIP2 HCRTR1 RIT2 KCNK3*
Synaptic signaling	20	0.012	*CACNG4 NMU NGF GLRA1 CACNG2 SLC17A8 SHISA9 SEZ6 SLC8A3 P2RX3 CSPG5 TNR SHC3 GJD2 GRID2IP ETV5 KCNIP2 HCRTR1 RIT2 KCNK3*
Behavior	19	0.004	*NGF TRH ABCC8 SEZ6 ASIC4 SEZ6L KCNK10 SLC8A3 NMU P2RX3 TNR HCRTR1 UNC79 GLRA1 SHC3 ALK SEZ6L2 PAX5 ETV5*
Circulatory system process	16	0.015	*TNNT1 ACE MYH7 FGFBP3 NOX4 NMU HCN4 ACTC1 CACNG4 SLC8A3 KCNIP2 CACNG2 COL4A3 KCNK3 KCNIP1 KCNJ11*
Blood circulation	15	0.027	*TNNT1 ACE MYH7 FGFBP3 NMU HCN4 ACTC1 CACNG4 SLC8A3 KCNIP2 CACNG2 COL4A3 KCNK3 KCNIP1 KCNJ11*
Heart process	14	0.001	*TNNT1 MYH7 NOX4 NMU HCN4 ACTC1 ACE CACNG4 SLC8A3 KCNIP2 CACNG2 KCNK3 KCNIP1 KCNJ11*
Heart contraction	13	0.002	*TNNT1 MYH7 NMU HCN4 ACTC1 ACE CACNG4 SLC8A3 KCNIP2 CACNG2 KCNK3 KCNIP1 KCNJ11*
Regulation of cytosolic calcium ion concentration	12	0.022	*PTGFR FAM155B TRPC3 CMKLR1 HCRTR1 CXCL13 C1QTNF1 SLC8A3 NMU GALR1 KCNK3 P2RX3*
Multicellular organismal signaling	11	0.002	*CACNG4 CACNG2 P2RX3 TNR HCN4 GLRA1 SLC8A3 KCNIP2 KCNK3 KCNIP1 KCNJ11*
Regulation of blood circulation	11	0.018	*ACE MYH7 NMU HCN4 CACNG4 SLC8A3 KCNIP2 CACNG2 KCNK3 KCNIP1 KCNJ11*

	Regulation of heart contraction	10	0.017	*MYH7 NMU HCN4 CACNG4 SLC8A3 KCNIP2 CACNG2 KCNK3 KCNIP1 KCNJ11*
	Cellular potassium ion transport	9	0.022	*KCNK10 KCNG4 KCNK3 KCNH7 KCNJ11 KCNIP2 HCN4 KCNIP1 ABCC8*
	Potassium ion transmembrane transport	9	0.022	*KCNK10 KCNG4 KCNK3 KCNH7 KCNJ11 KCNIP2 HCN4 KCNIP1 ABCC8*
	Cardiac conduction	8	0.012	*HCN4 CACNG4 SLC8A3 KCNIP2 CACNG2 KCNK3 KCNIP1 KCNJ11*
	Negative regulation of cellular response to growth factor stimulus	8	0.015	*DAND5 NOG SPRY4 SKOR2 CXCL13 CREB3L1 VWC2L EPN2*
	Adult behavior	8	0.015	*SEZ6 SEZ6L UNC79 GLRA1 TRH ALK SEZ6L2 PAX5*
	Cerebellar Purkinje cell layer development	5	0.003	*SEZ6 LHX5 SEZ6L SEZ6L2 SKOR2*
	Cerebellar cortex development	5	0.017	*SEZ6 LHX5 SEZ6L SEZ6L2 SKOR2*
	Regulation of fibroblast growth factor receptor signaling pathway	4	0.018	*SPRY4 CREB3L1 FGFBP3 NOG*
	Slow-twitch skeletal muscle fiber contraction	3	0.001	*TNNT1 MB MYH7*
	Twitch skeletal muscle contraction	3	0.003	*TNNT1 MB MYH7*
	Voluntary skeletal muscle contraction	3	0.003	*TNNT1 MB MYH7*
GO CC	Cation channel complex	11	0.010	*ABCC8 CACNG4 TRPC3 CACNG2 KCNG4 SHISA9 KCNIP2 HCN4 KCNIP1 KCNJ11 VWC2L*
	Integral component of plasma membrane	37	0.012	*ABCC8 CACNG4 NOX4 KCNK10 HCRTR1 TRPC3 GLRA1 CDHR1 GALR1 CACNG2 KCNG4 ALK KCNK3 KCNH7 SHISA9 KCNIP2 HCN4 C1QTNF1 KCNIP1 KCNJ11 SLC8A3 P2RX3 SLC16A10 CSPG5 MMP24 EDA2R SCARA5 VWC2L TMC3 NGFR COL17A1 ASIC4 PTGFR KLRC1 IL11RA SLC39A2 CMKLR1*
	Intrinsic component of plasma membrane	37	0.012	*ABCC8 CACNG4 NOX4 KCNK10 HCRTR1 TRPC3 GLRA1 CDHR1 GALR1 CACNG2 KCNG4 ALK KCNK3 KCNH7 SHISA9 SLC8A3 KCNIP2 HCN4 C1QTNF1 KCNIP1 KCNJ11 P2RX3 SLC16A10 CSPG5 MMP24 EDA2R SCARA5 VWC2L TMC3 NGFR COL17A1 ASIC4 PTGFR KLRC1 IL11RA SLC39A2 CMKLR1*
	AMPA glutamate receptor complex	4	0.012	*CACNG4 CACNG2 SHISA9 VWC2L*
	Ion channel complex	12	0.012	*ABCC8 CACNG4 TRPC3 CACNG2 KCNG4 SHISA9 KCNIP2 HCN4 KCNIP1 KCNJ11 GLRA1 VWC2L*
	Inward rectifying potassium channel	2	0.013	*ABCC8 KCNJ11*
	Neuron projection terminus	7	0.013	*SLC18A1 NMU P2RX3 MICAL1 GLRA1 ATCAY SLC17A8*

Plasma membrane part	52	0.013	*ABCC8 CACNG4 NOX4 KCNK10 HCRTR1 HIP1R BLK IL11RA TRPC3 GLRA1 CDHR1 GFRA1 GALR1 CACNG2 KCNG4 ALK KCNK3 KCNH7 SHISA9 ANK1 SLC8A3 KCNIP2 PTGIS HCN4 ACE LPO C1QTNF1 KCNIP1 KCNJ11 PRCD P2RX3 SLC16A10 CSPG5 MMP24 EDA2R LRP4 MEGF11 GJD2 SCARA5 VWC2L SAPCD2 SPRY4 TMC3 GRID2IP RAB31 NGFR COL17A1 ASIC4 PTGFR KLRC1 SLC39A2 CMKLR1*
Perisynaptic extracellular matrix	2	0.013	*TNR ACAN*
Synapse-associated extracellular matrix	2	0.013	*TNR ACAN*
Perisynaptic space	2	0.013	*TNR ACAN*
Transmembrane transporter complex	12	0.013	*ABCC8 CACNG4 TRPC3 CACNG2 KCNG4 SHISA9 KCNIP2 HCN4 KCNIP1 KCNJ11 GLRA1 VWC2L*
Transporter complex	12	0.013	*ABCC8 CACNG4 TRPC3 CACNG2 KCNG4 SHISA9 KCNIP2 HCN4 KCNIP1 KCNJ11 GLRA1 VWC2L*
Potassium channel complex	6	0.016	*ABCC8 KCNG4 KCNIP2 HCN4 KCNIP1 KCNJ11*
Cell body	15	0.024	*L1CAM SEZ6 CACNG4 SEZ6L SLC8A3 P2RX3 LRP4 RLBP1 GLRA1 RIT2 SEZ6L2 SLC17A8 KCNJ11 HIP1R ACTC1*
Collagen type IV trimer	2	0.024	*COL4A4 COL4A3*
Ionotropic glutamate receptor complex	4	0.024	*CACNG4 CACNG2 SHISA9 VWC2L*
Somatodendritic compartment	19	0.024	*NGF L1CAM SHISA9 MLPH SEZ6 CACNG4 SEZ6L SLC8A3 P2RX3 LRP4 GLRA1 RIT2 CACNG2 ATCAY SEZ6L2 SLC17A8 KCNIP1 KCNJ11 HIP1R*
Terminal bouton	4	0.024	*SLC18A1 NMU P2RX3 MICAL1*
Axon terminus	6	0.024	*SLC18A1 NMU P2RX3 MICAL1 GLRA1 SLC17A8*
Extracellular matrix component	4	0.024	*COL4A4 COL4A3 TNR ACAN*
Network-forming collagen trimer	2	0.024	*COL4A4 COL4A3*
Collagen network	2	0.024	*COL4A4 COL4A3*
Intrinsic component of postsynaptic membrane	6	0.024	*CACNG4 SLC8A3 CSPG5 GLRA1 CACNG2 SHISA9*
Integral component of postsynaptic membrane	6	0.024	*CACNG4 SLC8A3 CSPG5 GLRA1 CACNG2 SHISA9*
Intrinsic component of synaptic membrane	7	0.024	*CACNG4 SLC8A3 P2RX3 CSPG5 GLRA1 CACNG2 SHISA9*
Integral component of synaptic membrane	7	0.024	*CACNG4 SLC8A3 P2RX3 CSPG5 GLRA1 CACNG2 SHISA9*
Neurotransmitter receptor complex	4	0.026	*CACNG4 CACNG2 SHISA9 VWC2L*
Central element	2	0.026	*SYCE2 SYCE1*
Basement membrane collagen trimer	2	0.026	*COL4A4 COL4A3*

	Ion channel activity	18	0.000	*ABCC8 CACNG4 KCNK10 P2RX3 FAM155B TRPC3 GLRA1 CACNG2 KCNG4 KCNK3 KCNH7 KCNJ11 TMC3 KCNIP2 HCN4 ANO5 ASIC4 KCNIP1*
	Cation channel activity	15	0.000	*ABCC8 CACNG4 KCNK10 P2RX3 FAM155B TRPC3 CACNG2 KCNG4 KCNK3 KCNH7 KCNJ11 KCNIP2 HCN4 ASIC4 KCNIP1*
	Channel activity	18	0.000	*ABCC8 CACNG4 KCNK10 P2RX3 FAM155B TRPC3 GLRA1 CACNG2 KCNG4 KCNK3 KCNH7 KCNJ11 TMC3 KCNIP2 HCN4 ANO5 ASIC4 KCNIP1*
	Passive transmembrane transporter activity	18	0.000	*ABCC8 CACNG4 KCNK10 P2RX3 FAM155B TRPC3 GLRA1 CACNG2 KCNG4 KCNK3 KCNH7 KCNJ11 TMC3 KCNIP2 HCN4 ANO5 ASIC4 KCNIP1*
	Substrate-specific channel activity	18	0.000	*ABCC8 CACNG4 KCNK10 P2RX3 FAM155B TRPC3 GLRA1 CACNG2 KCNG4 KCNK3 KCNH7 KCNJ11 TMC3 KCNIP2 HCN4 ANO5 ASIC4 KCNIP1*
	Metal ion transmembrane transporter activity	18	0.000	*SLC5A2 ABCC8 SLC18A1 CACNG4 KCNK10 FAM155B TRPC3 SLC39A2 CACNG2 KCNG4 KCNK3 KCNH7 KCNJ11 KCNIP2 HCN4 ASIC4 SLC8A3 KCNIP1*
GO MF	Gated channel activity	15	0.000	*CACNG4 P2RX3 FAM155B GLRA1 CACNG2 KCNG4 KCNH7 KCNJ11 TMC3 KCNIP2 HCN4 ANO5 KCNK10 KCNK3 KCNIP1*
	Potassium channel activity	9	0.000	*ABCC8 KCNK10 KCNG4 KCNK3 KCNH7 KCNJ11 KCNIP2 HCN4 KCNIP1*
	Inorganic molecular entity transmembrane transporter activity	24	0.000	*SLC5A2 ABCC8 SLC18A1 CACNG4 KCNK10 P2RX3 FAM155B TRPC3 GLRA1 SLC39A2 CACNG2 KCNG4 KCNK3 SLC17A8 KCNH7 KCNJ11 TMC3 KCNIP2 HCN4 ANO5 ASIC4 SLC8A3 SLC16A10 KCNIP1*
	Ion gated channel activity	14	0.001	*CACNG4 P2RX3 FAM155B GLRA1 CACNG2 KCNG4 KCNH7 KCNJ11 KCNIP2 HCN4 ANO5 KCNK10 KCNK3 KCNIP1*
	Inorganic cation transmembrane transporter activity	19	0.001	*SLC5A2 ABCC8 SLC18A1 CACNG4 KCNK10 P2RX3 FAM155B TRPC3 SLC39A2 CACNG2 KCNG4 KCNK3 KCNH7 KCNJ11 KCNIP2 HCN4 ASIC4 SLC8A3 KCNIP1*
	Ion transmembrane transporter activity	24	0.001	*SLC5A2 ABCC8 SLC18A1 CACNG4 KCNK10 P2RX3 FAM155B TRPC3 GLRA1 SLC39A2 CACNG2 KCNG4 KCNK3 SLC17A8 KCNH7 KCNJ11 TMC3 KCNIP2 HCN4 ANO5 ASIC4 SLC8A3 SLC16A10 KCNIP1*

Voltage-gated ion channel activity	10	0.001	*CACNG4 CACNG2 KCNG4 KCNH7 KCNJ11 KCNIP2 HCN4 KCNK10 KCNK3 KCNIP1*
Potassium ion transmembrane transporter activity	9	0.001	*ABCC8 KCNK10 KCNG4 KCNK3 KCNH7 KCNJ11 KCNIP2 HCN4 KCNIP1*
Voltage-gated channel activity	10	0.001	*CACNG4 CACNG2 KCNG4 KCNH7 KCNJ11 KCNIP2 HCN4 KCNK10 KCNK3 KCNIP1*
Cation transmembrane transporter activity	19	0.002	*SLC5A2 ABCC8 SLC18A1 CACNG4 KCNK10 P2RX3 FAM155B TRPC3 SLC39A2 CACNG2 KCNG4 KCNK3 KCNH7 KCNJ11 KCNIP2 HCN4 ASIC4 SLC8A3 KCNIP1*
Transmembrane transporter activity	26	0.002	*SLC5A2 ABCC8 SLC18A1 CACNG4 KCNK10 P2RX3 FAM155B TRPC3 GLRA1 SLC39A2 CACNG2 KCNG4 KCNK3 SLC17A8 KCNH7 KCNJ11 TMC3 KCNIP2 HCN4 ANO5 SLC7A14 ASIC4 SLC8A3 SLC16A10 KCNIP1 SLC35F1*
Voltage-gated cation channel activity	8	0.003	*CACNG4 CACNG2 KCNG4 KCNH7 KCNJ11 KCNIP2 HCN4 KCNK3*
RNA polymerase II regulatory region sequence-specific DNA binding	20	0.004	*TFAP2B LHX5 BARHL1 PDX1 CREB3L1 NKX6-3 MYT1 ASCL5 TBX5 FOXF2 RORC ATOH8 ETV4 SCRT1 TLX1 CREB3L4 PAX5 ESRRG ETV5 SCRT2*
RNA polymerase II regulatory region DNA binding	20	0.004	*TFAP2B LHX5 BARHL1 PDX1 CREB3L1 NKX6-3 MYT1 ASCL5 TBX5 FOXF2 RORC ATOH8 ETV4 SCRT1 TLX1 CREB3L4 PAX5 ESRRG ETV5 SCRT2*
Monovalent inorganic cation transmembrane transporter activity	13	0.004	*SLC5A2 ABCC8 SLC18A1 KCNK10 KCNG4 KCNK3 KCNH7 KCNJ11 KCNIP2 HCN4 ASIC4 SLC8A3 KCNIP1*
Transporter activity	28	0.005	*SLC5A2 ABCC8 SLC18A1 CACNG4 CETP KCNK10 P2RX3 FAM155B TRPC3 GLRA1 SLC39A2 CACNG2 KCNG4 KCNK3 SLC17A8 KCNH7 KCNJ11 TMC3 KCNIP2 HCN4 STARD4 ANO5 SLC7A14 ASIC4 SLC8A3 SLC16A10 KCNIP1 SLC35F1*
Voltage-gated potassium channel activity	6	0.005	*KCNG4 KCNH7 KCNJ11 KCNIP2 HCN4 KCNK3*
DNA-binding transcription activator activity, RNA polymerase II-specific	13	0.007	*BARHL1 MYT1 NKX6-3 ETV4 FOXF2 TFAP2B TBX5 TLX1 CREB3L4 PAX5 ESRRG ETV5 CREB3L1*
MAP kinase tyrosine/serine/threonine phosphatase activity	3	0.007	*DUSP4 DUSP9 DUSP6*
Transcription regulatory region sequence-specific DNA binding	20	0.007	*TFAP2B LHX5 BARHL1 PDX1 CREB3L1 NKX6-3 MYT1 ASCL5 TBX5 FOXF2 RORC ATOH8 ETV4 SCRT1 TLX1 CREB3L4 PAX5 ESRRG ETV5 SCRT2*
MAP kinase phosphatase activity	3	0.009	*DUSP4 DUSP9 DUSP6*

	Extracellular matrix structural constituent conferring tensile strength	4	0.011	*COL9A1 COL17A1 COL4A4 COL4A3*
	Sequence-specific double-stranded DNA binding	20	0.012	*TFAP2B LHX5 BARHL1 PDX1 CREB3L1 NKX6-3 MYT1 ASCL5 TBX5 FOXF2 RORC ATOH8 ETV4 SCRT1 TLX1 CREB3L4 PAX5 ESRRG ETV5 SCRT2*
	DNA-binding transcription factor activity, RNA polymerase II-specific	30	0.018	*TFAP2B LHX5 BARHL1 ETV4 MYT1 ETV5 NKX6-3 SCRT1 TBX5 FOXF2 RORC CREB3L1 TLX1 PDX1 CREB3L4 PAX5 ESRRG ATRX SALL4 MNX1 ZNF208 INSM2 ATOH8 ZNF676 ZNF257 ZNF99 SCRT2 ASCL5 ZBTB8B SKOR2*
KEGG/ Reactome	Cardiac conduction	6	0.003	*CACNG2 CACNG4 KCNJ11 KCNK10 KCNK3 SLC8A3*
	NCAM1 interactions	3	0.004	*COL4A3 COL4A4 COL9A1*
	Muscle contraction	7	0.004	*ACTC1 CACNG2 CACNG4 KCNJ11 KCNK10 KCNK3 SLC8A3*
	Collagen chain trimerization	4	0.004	*COL17A1 COL4A3 COL4A4 COL9A1*
	Adrenergic signaling in cardiomyocytes	7	0.004	*ACTC1 CACNG2 CACNG4 CREB3L1 CREB3L4 MYH7 SLC8A3*
	Assembly of collagen fibrils and other multimeric structures	4	0.004	*COL17A1 COL4A3 COL4A4 COL9A1*
	Collagen biosynthesis and modifying enzymes	4	0.004	*COL17A1 COL4A3 COL4A4 COL9A1*
	RAF-independent MAPK1/3 activation	3	0.004	*DUSP4 DUSP6 DUSP9*
	Protein digestion and absorption	6	0.005	*COL17A1 COL4A3 COL4A4 COL9A1 SLC16A10 SLC8A3*
	Effects of PIP2 hydrolysis	3	0.005	*DGKG DGKK TRPC3*
	Platelet calcium homeostasis	3	0.005	*P2RX3 SLC8A3 TRPC3*
	Insulin secretion	5	0.005	*ABCC8 CREB3L1 CREB3L4 KCNJ11 PDX1*
	Cardiac muscle contraction	5	0.005	*ACTC1 CACNG2 CACNG4 MYH7 SLC8A3*
	Hypertrophic cardiomyopathy (HCM)	6	0.005	*ACE ACTC1 CACNG2 CACNG4 MYH7 SLC8A3*
	Signaling by NTRK1 (TRKA)	4	0.006	*DUSP4 DUSP6 NGF SHC3*
	Dilated cardiomyopathy (DCM)	5	0.007	*ACTC1 CACNG2 CACNG4 MYH7 SLC8A3*
	Collagen formation	4	0.007	*COL17A1 COL4A3 COL4A4 COL9A1*
	NCAM signaling for neurite out-growth	3	0.009	*COL4A3 COL4A4 COL9A1*
	Signaling by NTRKs	4	0.009	*DUSP4 DUSP6 NGF SHC3*
	Potassium Channels	5	0.009	*ABCC8 HCN4 KCNJ11 KCNK10 KCNK3*
	Negative regulation of MAPK pathway	3	0.011	*DUSP4 DUSP6 DUSP9*
	Type II diabetes mellitus	3	0.018	*ABCC8 KCNJ11 PDX1*
	ECM-receptor interaction	4	0.019	*COL4A3 COL4A4 COL9A1 TNR*

Cocaine addiction	3	0.020	*CREB3L1 CREB3L4 SLC18A1*
Prostate cancer	4	0.024	*CDKN1A CREB3L1 CREB3L4 ETV5*
Signaling by PDGF	3	0.025	*COL4A3 COL4A4 COL9A1*
Glycerolipid metabolism	3	0.031	*DGKG DGKK PNPLA3*
Transport of inorganic cations/anions and amino acids/oligopeptides	3	0.031	*SLC16A10 SLC17A8 SLC8A3*
Cortisol synthesis and secretion	3	0.034	*CREB3L1 CREB3L4 KCNK3*
Amphetamine addiction	3	0.038	*CREB3L1 CREB3L4 SLC18A1*
Integrin cell surface interactions	3	0.044	*COL4A3 COL4A4 COL9A1*
Platelet homeostasis	3	0.044	*P2RX3 SLC8A3 TRPC3*
Degradation of the extracellular matrix	3	0.044	*COL4A3 COL4A4 MMP24*

© 2020 by the authors. Licensee MDPI, Basel, Switzerland. This article is an open access article distributed under the terms and conditions of the Creative Commons Attribution (CC BY) license (http://creativecommons.org/licenses/by/4.0/).
